# HNF4α contributes to hepatic CAR dysfunction in polymicrobial sepsis

**DOI:** 10.3389/fimmu.2025.1625104

**Published:** 2025-08-19

**Authors:** Céline Van Dender, Steven Timmermans, Maxime Roes, Madeleine Hellemans, Elise Moens, Louise Nuyttens, Maarten Claes, Bart Roman, Karolien De Bosscher, Jolien Vandewalle, Claude Libert

**Affiliations:** ^1^ Center for Inflammation Research, Vlaams Instituut voor Biotechnologie (VIB), Ghent, Belgium; ^2^ Department of Biomedical Molecular Biology, Ghent University, Ghent, Belgium; ^3^ Center for Medical Biotechnology, Vlaams Instituut voor Biotechnologie (VIB), Ghent, Belgium; ^4^ Department of Biomolecular Medicine, Ghent University, Ghent, Belgium; ^5^ Research Group SynBioC, Department of Green Chemistry and Technology, Faculty of Bioscience Engineering, Ghent University, Ghent, Belgium

**Keywords:** HNF4α, CAR dysfunction, liver, sepsis, acute phase response, metabolism

## Abstract

The constitutive androstane receptor (CAR), encoded by the *Nr1i3* gene, is a nuclear receptor mainly expressed in the liver, where it regulates (xenobiotic) drug and bile acid metabolism, bilirubin clearance and energy homeostasis. CAR has emerged as a promising therapeutic target for diabetes, fatty liver disease and alcoholic liver disease, but it has barely been investigated in the context of sepsis. Since alterations in drug metabolism have been observed in sepsis patients, who may also exhibit increased serum bilirubin and bile acid levels, we hypothesize that CAR function may be impaired during sepsis. Here, we demonstrate that CAR loses its function in the liver during sepsis, as evidenced by a diminished response to its agonist TCPOBOP. We show that *Nr1i3* mRNA transcription is reduced, mediated by decreased HNF4α binding to the *Nr1i3* promoter and by downregulation of *Ppara* expression. Additionally, we show that CAR DNA binding is impaired, and we propose that HNF4α may regulate chromatin accessibility of CAR binding sites in sepsis. CAR loss-of-function further causes the downregulation of genes involved in monocarboxylic acid, fatty acid, and xenobiotic metabolism, but induces a hepatic acute phase response, which is beneficial for liver regeneration. However, CAR inhibition with CINPA1 increases sepsis lethality, associated with the further downregulation of these metabolic genes, increased upregulation of the acute phase response, but persistent downregulation of proliferation markers in the liver. Altogether, our study highlights the importance of CAR in sepsis with respect to hepatic metabolism, liver regeneration and survival. Nevertheless, CAR is unlikely to serve as a viable therapeutic target in sepsis, given its rapid downregulation and the lack of a survival benefit from TCPOBOP treatment. Instead, targeting upstream regulators such as HNF4α may represent a more effective approach.

## Introduction

Sepsis has an annual prevalence of 49 million and a mortality of 11 million, making it one of the most unmet medical needs of today (1). It is described as a life-threatening organ dysfunction caused by a dysregulated host response to infection (2). Inflammation was previously thought to be the primary cause of death, but many immunomodulating agents have failed in the clinic, limiting current sepsis therapies to antibiotics, vasopressors, fluid resuscitation, and organ support (3, 4).

Recent evidence highlights a significant role of metabolic dysregulation in sepsis pathogenesis, which is characterized by tachycardia, fever, tachypnoea, immune activation, complement and coagulation activation, and the acute phase response (APR) (5). Since sepsis patients display severely reduced food intake, these features impose supraphysiological energy demands, triggering a starvation response (6). During such response, glycogen is depleted to release glucose, muscle proteins are broken down into amino acids, and lipids from white adipose tissue are hydrolyzed to release free fatty acids (FFAs) and glycerol. However, due to the failure of peroxisome proliferator-activated receptor α (PPARα) and the glucocorticoid receptor (GR) in the liver, which regulate FFA import, oxidation and ketogenesis, and gluconeogenesis, respectively, these molecules are not further metabolized, leading to their (toxic) accumulation and to energy shortage (7, 8).

We recently published that hepatocyte nuclear factor 4 α (HNF4α) is a major protective factor and one of the top upstream regulators in acute murine septic peritonitis, as well as in pigs and in mice with a humanized liver (9). HNF4α belongs to the nuclear receptor superfamily and maintains liver identity by regulating numerous genes involved in lipid, carbohydrate, bile acid, and xenobiotic metabolism (10). HNF4α upregulates *Ppara* expression by binding to the DR1 element within its gene promoter but also facilitates GR binding to its liver-specific elements by promoting chromatin opening (11–13). The progressive loss of HNF4α activity in sepsis is associated with diminished chromatin binding of HNF4α, thereby reducing H3K27 acetylation and chromatin accessibility and causing the downregulation of many genes coding for proteins, among which nuclear receptors such as PPARα. Targeting HNF4α with the agonist N-trans caffeoyl tyramine (NCT) provided protection in sepsis, both prophylactically and therapeutically, by enhancing HNF4α chromatin binding and promoting the expression of its target genes. Additionally, NCT reduced lipid accumulation in the blood and liver, improved the hepatic APR, and decreased inflammation and organ dysfunction (9). Although HNF4α loss-of-function in sepsis affects the chromatin state, PPARα activity, lipid metabolism, and the APR, its impact on the activity of other nuclear receptors remains unclear.

An interesting nuclear receptor, whose expression is mainly regulated by HNF4α and has barely been investigated in sepsis, is the constitutive androstane receptor (CAR), encoded by the *Nr1i3* gene (14). CAR is primarily expressed in hepatocytes, where it plays a major role in endobiotic and xenobiotic metabolism, as well as drug detoxification, by controlling the expression of drug-metabolizing enzymes, including cytochrome P450 (CYP) enzymes, and various cellular metabolite transporters (15). It also contributes to energy homeostasis by regulating carbohydrate and lipid metabolism (16, 17). Furthermore, CAR is essential for proper liver regeneration, as shown in models of partial hepatectomy and liver injury induced by 3,5-diethoxycarbonyl-1,4-dihydrocollidine (DDC), where it regulates the proliferation of hepatocytes and hepatic progenitor cells (18–20).

In contrast to other nuclear receptors, which comprise an N-terminal A/B domain with activation function-1 (AF1), a DNA-binding domain, a hinge region, a ligand-binding domain with activation function-2 (AF2) and a C-terminal F domain, CAR lacks an A/B domain with AF1 and can exert ligand-independent activity by mimicking an active AF2 conformation (21). This conformation allows CAR to interact with coactivators in the absence of a ligand (22). However, although CAR is constitutively activated in immortalized cell lines, it is not in the liver *in vivo* or in primary hepatocytes (23). This is because the endogenous CAR ligands, androstenol and androstanol, act as repressors of the constitutive activity of CAR, maintaining a low basal level of activity, and are considered inverse agonists (24). Additionally, in the original concept of CAR activation, CAR is retained in the cytoplasm within a complex comprising HSP90, CCRP, and PPP1R16A, stabilized by HSP70, when phosphorylated at threonine 38 (position 48 in mice) by protein kinase C (PKC) (25–27). Dephosphorylation of CAR by protein phosphatase 2A (PP2A) is required to facilitate its translocation to the nucleus (28, 29). CAR has also been reported to form homodimers in the cytoplasm, which mask the PP2A site and prevent its nuclear translocation (30). It was proposed that CAR can be activated through both ligand-dependent and ligand-independent mechanisms, and that these are initiated by the nuclear translocation of CAR from the cytoplasm (26). CAR ligands can act either directly (e.g., TCPOBOP and CITCO) or indirectly (e.g., phenobarbital) by inhibiting EGFR, thereby activating PP2A and promoting CAR dephosphorylation (31–33). Endogenous CAR activators, however, remain poorly characterized. Candidates include diindoles from commensal bacteria metabolites, 17beta-estradiol and estrone (34, 35). However, a more recent study suggests that CAR1 (the reference isoform) is constitutively present in the nucleus under basal conditions, although a small fraction remains cytoplasmic and translocates to the nucleus upon activation. Nevertheless, nuclear localization alone is insufficient for its activation, with phosphorylation and dephosphorylation potentially playing a regulatory role (36).

It has already been shown that hepatic *Nr1i3* mRNA is downregulated starting 4h after LPS injection in mice, associated with reduced expression of CAR target gene *Cyp2b10* (37). Furthermore, many hepatic CYP enzymes and drug transporters are downregulated in septic mice and pigs, and alternations in drug metabolism have been observed in sepsis patients in the clinic (38, 39). CAR also plays a key role in the response to starvation, a critical feature in sepsis. This is evident in CAR knockout (KO) mice, which lose twice as much weight as littermate controls when subjected to a 40% caloric restriction diet for 12 weeks, likely due to an attenuated reduction in thyroid hormone levels (40). Together, these data suggest an impaired CAR function during sepsis progression. In this study, we aim to investigate whether CAR function is impaired in acute sepsis, identify the mechanisms responsible for this impairment, and determine the consequences for hepatic metabolism and survival.

We use the cecal ligation and puncture (CLP) mouse model, which is considered the gold standard for polymicrobial sepsis, to study hepatic CAR function during sepsis. We found that CAR expression is strongly reduced, suggesting a loss-of-function following sepsis, and causing the downregulation of CAR-dependent genes involved in monocarboxylic acid, fatty acid, and drug metabolism, but inducing a beneficial hepatic APR. However, when CAR function is further inhibited by CINPA1 during sepsis, lethality is increased, which is associated with the further downregulation of these metabolic genes, increased systemic inflammation and greater organ damage. We also show that HNF4α regulates CAR activity both by controlling its transcription and by modulating its DNA binding capacity via chromatin remodeling, both of which are disrupted in CLP.

## Materials and methods

### Mice

Male C57BL/6J mice were ordered from Janvier (Le Genest-St. Isle, France). Mutant Hnf4a^fl/fl^ mice were generated by Dr. Frank Gonzalez (NIH, Bethesda, USA) and formally called B6.129X1(FVB)-*Hnf4a^tm1.1Gonz^*/J (41). Exons 4 and 5 were flanked by loxP sites using ES cell technology. The mice had been backcrossed into C57BL/6J background and were provided by Dr. Iannis Talianidis (University of Crete, Heraklion, Greece), by courtesy of Dr. Frank Gonzalez, and were under protection of an MTA. Subsequently, we crossed these mice with AlbCreERT2^Tg/+^ mice, which were kindly provided by Dr. D. Metzger & Dr. P. Chambon (Igbmc, France) (42). The humanized liver mice were generated by Prof. Philip Meuleman (UGhent, Belgium) as described in (43). Briefly, the mice were generated by transplanting homozygous Alb-uPA^+/+^-SCID mice (which suffer from spontaneous and chronic death of hepatocytes) with 0.7 x 10^6^ cryopreserved primary human hepatocytes (donor C342, Lonza), via intrasplenic injection. The human albumin concentration in plasma was determined 6 weeks after transplantation by ELISA (Bethyl Laboratories, Montgomery, Texas, United States) and was used as a marker of liver chimerism.

All mice were housed in individually ventilated cages (IVC) at standard housing conditions (22°C, 14/10h light/dark cycle) with food (chow diet consisting of 18% proteins, 4.5% fibers, 4.5% fat, 6.3% ashes, Provimi Kliba SA) and water *ad libitum*. The IVC were kept in a specific pathogen free facility. Both male and female offspring was used between 8 and 20 weeks. All experiments were approved by the institutional ethics committee for animal welfare of the Faculty of Sciences, Ghent University, Belgium (EC2024-059). The methods were carried out in accordance with the relevant guidelines and regulations.

Mice were subjected to CLP operation (44). Briefly, mice were anesthetized with isoflurane, followed by a 1 cm incision in the abdominal skin. The caecum was ligated for 75% and punctured twice with a 21-gauche needle, which allows the cecal content to leak into the abdomen and cause a systemic infection. The abdominal musculature and skin were closed with simple running sutures and metallic clips, respectively. Sham mice underwent the same procedure without ligating and puncturing the caecum. During lethality studies, mice were injected intraperitoneally (IP) with broad-spectrum antibiotics (25 mg/kg ceftriaxone and 12.5 mg/kg metronidazole) in phosphate buffered saline (PBS) 8h and 24h after CLP. Rectal BT was measured 3 times a day and mice were euthanized when their BT dropped below 28°C (= human endpoint).

To induce hepatic HNF4α depletion, Hnf4a^fl/fl^;AlbCreERT2^Tg/+^ and Hnf4a^fl/fl^;AlbCreERT2^+/+^ mice were IP injected with 1 mg tamoxifen in a 1:8 ethanol:oil solution for five consecutive days. HNF4α protein was completely depleted three days after the final injection (9). To investigate CAR activity, the CAR agonist TCPOBOP (12.5 mg/kg, dissolved in 50% corn oil and 50% DMSO) was administered via IP injection in non-operated wild-type mice, in Hnf4a^Liver-i-KO^ mice three days after the last tamoxifen injection, and in septic mice 6h or 24h after sham or CLP surgery. For survival studies, TCPOBOP (3 mg/kg, dissolved in 90% corn oil and 10% DMSO) was administered via IP injection or oral gavage for four consecutive days. To assess the functional consequences of CAR loss-of-function, the CAR inhibitor CINPA1 (12.5 mg/kg, dissolved in 90% corn oil and 10% DMSO) was administered via IP injection for four consecutive days. Additionally, the HNF4α agonist NCT was administered via IP injection for 7 days, with 1 injection during the first 3 three days, 2 injections during the following three days, and 1 injection on the day of CLP, at a dose of 200 mg/kg, dissolved in DMSO. Mice co-treated with NCT and CINPA1 also received 10 doses of NCT (200 mg/kg, dissolved in 2-hydroxypropyl-β-cyclodextrin (25 mg/mL in PBS)), including 2 additional injections during the first 2 days after CLP. They also received 4 doses of CINPA1 (12.5 mg/kg, dissolved in 90% corn oil and 10% DMSO), with 1 additional injection given 1 day post-CLP. NCT was synthetized by SynBioC (Dr. Bart Roman).

### Liver transcriptomic analysis

The liver was isolated and stored in RNAlater (Life Technologies Europe), after which RNA was extracted using the Aurum Total RNA Mini kit (Bio-Rad) according to the manufacturer’s-protocol. RNA concentration was measured, and quality was assessed using the Nanodrop 1000 (Thermo Scientific).

#### Real-time qPCR

1000 ng of RNA was reverse transcribed into cDNA using the iScript cDNA Synthesis kit (Roche). The cDNA was diluted 10-fold in nuclease-free water prior to qPCR analysis, which was performed using the Light Cycler 480 system (Roche) and Sensifast Bioline Mix (Bio-Line). Samples were loaded in duplicate, and housekeeping genes *Gapdh*, *Hprt* and/or *Rpl* for mouse genes and *CYPB* and *UBC* for human genes were included to normalize target gene expression. The qPCR primers and associated sequences are listed in **Supplementary Tables S1** and **S2**. qPCR data were analyzed using qbase+ software (Biogazelle, Gent, Belgium).

#### RNA sequencing

##### Illumina stranded mRNA Prep, ligation

RNA concentration and purity were determined spectrophotometrically using the Nanodrop ND-8000 (Nanodrop Technologies) and RNA integrity and concentration were assessed using a Fragment Analyzer Standard Sensitivity RNA kit (Agilent).

Per sample, an amount of 1000ng of total RNA was used as input. Using the Illumina Stranded mRNA Sample Prep Kit (protocol version: # 1000000124518 v03, June 2022), poly-A containing mRNA molecules were purified from the total RNA input using poly-dT oligo-attached magnetic beads. The purified mRNA was fragmented, and in a reverse transcription reaction using random primers and Actinomycin D, RNA was converted into first strand cDNA, and subsequently into double-stranded cDNA in a second strand cDNA synthesis reaction using dUTP to achieve strand specificity. The cDNA fragments were extended with a single ‘A’ base to the 3’ ends of the blunt-ended cDNA fragments, after which pre-index anchors were ligated preparing the fragments for dual indexing. Anchor-ligated fragments were then purified using magnetic beads. Finally, enrichment PCR was carried out to enrich those DNA fragments that have anchor-ligated DNA fragments and to add indexes and primer sequences for cluster generation.

Purified dual-indexed sequence-libraries of each sample were equimolarly pooled and sequenced on Element Biosciences AVITI [2x75 Cloudbreak High kit, single read 100 (101–10–10–0)] at the VIB Nucleomics Core (https://nucleomicscore.sites.vib.be/en).

#### Data analysis

Single-end 100 bp reads were mapped to the mouse (mm39) reference genome using STAR (45). Multimapping reads were excluded from the data processing. Differential expression analysis was conducted using the DESeq2 package (46), with the FDR set at 5%. RNA-Seq data were analyzed with Metascape (47) and ClustVis (48).

### Western blot analysis

Total protein was isolated from snap-frozen livers using RIPA lysis buffer, supplemented with a protease inhibitor cocktail (Roche). Protein concentration was determined using the Bradford assay. Protein samples containing 30 µg of protein, along with loading dye, were separated by electrophoresis on an 8% sodium dodecyl sulfate (SDS)-polyacrylamide gel and transferred to a nitrocellulose membrane (0.45 µm pore size) by blotting. The membrane was blocked with a ½ dilution of Starting Block/PBST 0,1% (Thermo Fisher Scientific) and incubated overnight at 4°C with primary antibodies against CAR (1:667; ab186869, Abcam), (Ser/Thr/Tyr)-phospho (1:1000; ab15556, Abcam), CYP2B10 (1:1000; AB9916, Sigma-Aldrich), HNF4α (1:1000; PP-H1415-0C, R&D systems), and β-actin (1:5000; BA3R, Thermo Fisher Scientific) as an internal control. After washing with PBST 0,1%, the membrane was incubated for 1h at room temperature (RT) with either Amersham ECL anti-mouse antibody (1:2000; GENA931, GE Healthcare Life Sciences), Amersham ECL anti-rabbit antibody (1:2000; GEN934, GE Healthcare Life Sciences), or VeriBlot (1:2000; ab131366, Abcam) for the immunoprecipitation (IP) samples. The membrane was then washed again with PBST 0,1%, and immunoreactive bands were visualized and quantified using an Amersham Imager 600 (GE Healthcare Life Sciences) and the WesternBright Quantum kit (Advansta).

### Immunoprecipitation

Since no antibody for phospho-CAR is available, we immunoprecipitated CAR from total liver lysates and used a general anti-phospo antibody for Western blot analysis. Total protein was isolated from 50 mg of snap-frozen liver tissue using RIPA lysis buffer, supplemented with a protease inhibitor cocktail (Roche) and phosphatase inhibitors (20 mM glycerophosphate, 1 mM NaF, 1 mM Na_3_VaO_4_). Meanwhile, Dynabeads™ protein A (Thermo Fisher Scientific) were washed three times with RIPA, then blocked with RIPA containing BSA for 10 min at RT. To reduce nonspecific binding to the beads, protein lysates were precleared by incubation with the blocked beads for 1h at 4°C. Protein concentration was then measured using the Bradford assay, and input samples were collected. The precleared lysates (containing 300 µg of protein) were incubated with primary antibodies against CAR (4.8 µg/IP sample; ab186869, Abcam) or rabbit GFP-IgG (4.8 µg/IP sample; AB3080, Sigma-Aldrich) as an internal control for 1h at 4°C. We worked with pooled IgG samples, meaning that all replicates for each condition were combined for incubation with the IgG antibody. The IP samples were then incubated with the BSA-blocked beads for 2h at 4°C. Afterward, bead-antibody-protein complexes were washed 5 times with RIPA, then lysed in RIPA with loading dye for 5 min at 95°C.

### Mass spectrometry

#### Sample preparation

Per mg snap-frozen liver tissue, 10 µl lysis buffer (5% SDS and 50 mM triethylammonium bicarbonate (TEAB), pH 8.5) and 1 stainless steel 5 mm bead was added for homogenization using a Qiagen Tissue Lyser II. The samples were placed in cooled adapter racks and homogenized for 4 times 2 minutes at 20 Hz. Of each homogenate, 100 µl was transferred to a 96-well PIXUL plate and sonicated with a PIXUL Multisample sonicator (Active Motif) for 10 minutes with default settings (Pulse 50 cycles, PRF 1 kHz, Burst Rate 20 Hz). After centrifugation of the samples for 15 minutes at maximum speed at RT to remove insoluble components, the protein concentration was measured by bicinchoninic acid (BCA) assay (Thermo Scientific) and from each sample 100 µg of protein was isolated to continue the protocol. Proteins were reduced and alkylated by addition of 10 mM Tris(2-carboxyethyl)phosphine hydrochloride and 40 mM chloroacetamide and incubation for 10 minutes at 95°C in the dark. Phosphoric acid was added to a final concentration of 1.2% and subsequently samples were diluted 7-fold with binding buffer containing 90% methanol in 100 mM TEAB, pH 7.55. The samples were loaded on the 96-well S-Trap™ plate (Protifi), placed on top of a deepwell plate, and centrifuged for 2 min at 1,500 x g at RT. After protein binding, the S-trap™ plate was washed three times by adding 200 µl binding buffer and centrifugation for 2 min at 1,500 x g at RT. A new deepwell receiver plate was placed below the 96-well S-Trap™ plate and 50 mM TEAB containing trypsin (1/100, w/w) was added for digestion overnight at 37°C. Using centrifugation for 2 min at 1,500 x g, peptides were eluted three times, first with 80 µl 50 mM TEAB, then with 80 µl 0.2% formic acid (FA) in water and finally with 80 µl 0.2% FA in water/acetonitrile (ACN) (50/50, v/v). Eluted peptides were dried completely by vacuum centrifugation. Samples were dissolved in 100 µl 0.1% TFA and desalted on a reversed phase (RP) C18 OMIX tip (Agilent). The tip was first washed 3 times with 100 µl pre-wash buffer [0.1% TFA in water/ACN (20:80, v/v)] and pre-equilibrated 5 times with 100 µl of wash buffer (0.1% TFA in water) before the sample was loaded on the tip. After peptide binding, the tip was washed 3 times with 100 µl of wash buffer and peptides were eluted twice with 100 µl elution buffer (0.1% TFA in water/ACN (40:60, v/v)). The combined elutions were transferred to HPLC inserts and dried in a vacuum concentrator.

#### MS analysis

Peptides were re-dissolved in 20 µl loading solvent A [0.1% trifluoroacetic acid in water/acetonitrile (ACN) (99.5:0.5, v/v)] of which 2 µl of sample was injected for LC-MS/MS analysis on an Ultimate 3000 ProFlow nanoLC system in-line connected to a Q Exactive HF mass spectrometer (Thermo) equipped with pneu-Nimbus dual ion source (Phoenix S&T). Trapping was performed at 20 μl/min for 2 min in loading solvent A on a PepMap™ Neo Trap column (Thermo scientific, 300 μm internal diameter (I.D.), 5 μm beads). The peptides were separated on an 250 mm Odyssey Ultimate, 1.7µm C18, 75 µm inner diameter (Ionopticks) kept at a constant temperature of 45°C. Peptides were eluted by a gradient reaching 26,4% MS solvent B (0.1% FA in acetonitrile) after 75 min, 44% MS solvent B at 95 min, 56% MS solvent B at 100 min followed by a 5-minutes wash at 56% MS solvent B and re-equilibration with MS solvent A (0.1% FA in water). The flow rate was set to 250 nl/min.

The mass spectrometer was operated in data-independent mode, automatically switching between MS and MS/MS acquisition. Full-scan MS spectra ranging from 375–1500 m/z with a target value of 5E6, a maximum fill time of 50 ms and a resolution of 60,000 were followed by 30 quadrupole isolations with a precursor isolation width of 10 m/z for HCD fragmentation at an NCE of 30% after filling the trap at a target value of 3E6 for maximum injection time of 45 ms. MS2 spectra were acquired at a resolution of 15,000 in the Orbitrap analyzer. The isolation intervals ranging from 400–900 m/z, without overlap, were created with the Skyline software tool. The polydimethylcyclosiloxane background ion at 445.120028 Da was used for internal calibration (lock mass) and QCloud has been used to control instrument longitudinal performance during the project (49, 50).

#### Data analysis

Analysis of the MS data was performed in DiaNN (version 1.9.2) (51). Precursor false discovery rate was set at 1%. Spectra were searched against the Mus musculus protein sequences in the Uniprot database (database release version of 2024_01), containing 21,701 sequences (www.uniprot.org). Enzyme specificity was set as C-terminal to arginine and lysine, also allowing cleavage at proline bonds with a maximum of 1 missed cleavage. Variable modifications were set to oxidation of methionine residues and acetylation of protein N-termini while fixed modification was set to carbamidomethylation of cystein residues. Matching between runs was enabled. Mainly default settings were used, except for the addition of a 400–700 m/z precursor mass range filter and MS1 and MS2 mass tolerance was set to 10.0 and 20 ppm respectively.

Further data analysis of the results was performed with an in-house script in the R programming language. Protein expression matrices were prepared as follows: the DIA-NN main report output table was filtered at a precursor and protein library q-value cut-off of 1% and only proteins identified by at least one proteotypic peptide were retained. After pivoting into a wide format, iBAQ intensity columns were then added to the matrix using the DIAgui’s R package get_IBAQ function (52). PG MaxLFQ intensities were log2 transformed and replicate samples were grouped. Proteins with less than 3 valid values in at least one group were removed and missing values were imputed from a normal distribution centered around the detection limit [package DEP (53)] leading to a list of 3,321 quantified proteins in the experiment, used for further data analysis. To compare protein abundance between pairs of sample groups (CLP vs Sham sample groups), statistical testing for differences between two group means was performed, using the package limma (54). Statistical significance for differential regulation was set to a false discovery rate (FDR) of < 0.05 and |log2FC| = 1.

### Chromatin immunoprecipitation-qPCR

ChIP was performed on 50 mg of snapfrozen liver tissue, homogenized in PBS, and crosslinked with PBS + 2% formaldehyde for 20 min at RT. The crosslinking was quenched with 1M glycine (final concentration: 0.125M) for 10 min at RT. Liver homogenates were washed twice with PBS and then lysed with a buffer containing 0.1% SDS and 1% Triton-X-100. The liver lysates were sonicated (Bioruptor, Diagenode) at 4°C for 30 cycles (30s ON, 30s OFF) at high settings to yield 200–800 bp DNA fragments. Cell debris was removed by centrifugation at 10,000g for 5 min at 4°C, and input samples were collected. During sonication, nProtein G Sepharose™ 4 Fast Flow beads (Cytiva) were washed three times with incubation buffer containing 0.15% SDS and 1% Triton-X-100, then blocked with incubation buffer containing BSA for at least 2h at 4°C. Afterwards, sonicated samples were incubated with primary antibodies against HNF4α (5 µg/IP sample; PP-H1415-0C, Bio-Techne) or mouse IgG (5 µg/IP sample; sc-2025, SantaCruz) as an internal control for 2h at 4°C. These samples were then incubated with the BSA-blocked beads overnight at 4°C. The day after, bead-antibody-DNA complexes were washed twice with wash buffer containing 0.1% SDS, 0.1% sodium-deoxycholate, 1% Triton-X-100, 0.15M NaCl, 1 mM EDTA, and 20 mM HEPES; once with wash buffer containing 0.1% SDS, 0.1% sodium-deoxycholate, 1% Triton-X-100, 0.5M NaCl, 1 mM EDTA, and 20 mM HEPES; once with wash buffer containing 0.25M LiCl, 0.5% sodium-deoxycholate, 0.5% NP-40, 0.15M NaCl, 1 mM EDTA, and 20 mM HEPES; and twice with wash buffer containing 1 mM EDTA and 20 mM HEPES at 4°C for 5 min. Chromatin was eluted by incubating the IP samples with elution buffer (1% SDS, 0.1M NaHCO_3_) 20 min at RT. Both IP and input samples were first incubated with proteinase K and RNase A for 2 hours at 56°C, then overnight at 65°C for reverse crosslinking. The DNA was purified in 50 µL elution buffer according to the manufacturer’s protocol by using the QIAquick PCR purification kit (Qiagen).

qPCR was performed on 2 µL of purified DNA from IP and input samples using the Light Cycler 480 system (Roche) and Sensifast Bioline Mix (Bio-Line) to assess HNF4α binding at the *Nr1i3* promoter. qPCRs were carried out in duplicate. Sequences of the qPCR primers used: Forward primer (5’-3’) AACCAACACTTCTCGGGCA and Reverse primer (5’-3’) GCCTCTAGGTATCCTCGGTG. Results after immunoprecipitation were subtracted from the input and expressed as relative enrichment to the negative IgG control.

### Nuclear protein lysates

Nuclear protein lysates were prepared from 40 mg of snap-frozen liver tissue. First, the liver was homogenized in PBS supplemented with a protease inhibitor cocktail (Roche). Next, nuclei were isolated using a lysis buffer containing 0.5% NP-40 and 0.25% Triton-X100, with a protease inhibitor cocktail (Roche). The isolated nuclei were then washed and lysed in a buffer containing 0.5% N-lauroylsarcosine and 0.1% Na-Deoxycholate, again supplemented with a protease inhibitor cocktail (Roche). Finally, the samples were sonicated using a PIXUL Multisample sonicator (Active Motif) for 15 minutes with default settings (Pulse 50 cycles, PRF 1 kHz, Burst Rate 20 Hz). Protein concentration was determined using the Bradford assay.

### Biochemical analysis

Analysis of organ damage markers, ALT, AST and LDH, in plasma was performed at the University Hospital of Ghent. Plasma SAA protein levels (ICL lab; E-90SAA), plasma IL6 levels (Thermofisher; 88-7064-88), hepatic CAR protein levels (Antibodies-online; ABIN6969015), and the DNA binding activity of CAR (Abbexa; abx596559) in liver nuclear protein lysates (containing 50 µg of protein) were measured by ELISA according to the manufacturer’s instructions.

### Statistical analysis

Plasma IL6 levels and qPCR data were log-transformed to obtain normal distribution. Normality was assessed using a normality test in Prism 9.0 (GraphPad Software, Inc), which was also used primarily for plotting. When comparing two group means, an unpaired student’s t-test was used. If the standard deviation (SD) between groups differed significantly, Welch’s correction was applied. For comparisons involving more than two groups, P-values were analyzed using a one-way ANOVA with the uncorrected Fisher’s LSD multiple comparisons test. Two-way ANOVA with either the uncorrected Fisher’s LSD or Tukey’s multiple comparisons test was used for analyses involving a second variable. Kaplan-Meier survival curves were compared using the log-rank test. XY-plots were analyzed using simple linear regression. P-values of < 0.05 were considered statistically significant. ∗∗∗∗P ≤ 0.0001, ∗∗∗P ≤ 0.001, ∗∗P ≤ 0.01, ∗P ≤ 0.05, ns: not significant. n represents the number of biological replicates used to generate the data, determined based on previous experiments. Each experiment shown in the main figures was repeated at least twice. Data are represented as dot plots with bars indicating the mean ± SD or SEM. Additional statistical details can be found in the figures and/or figure legends. To mitigate the influence of sex- or age-related factors, an equal number of animals of each sex and similar age were assigned to each treatment group. Treatments were also distributed across different cages. Data analysis was performed blindly to avoid subjective bias.

## Results

### Sepsis impairs CAR transcriptional activity in the liver

**Figure 1:**
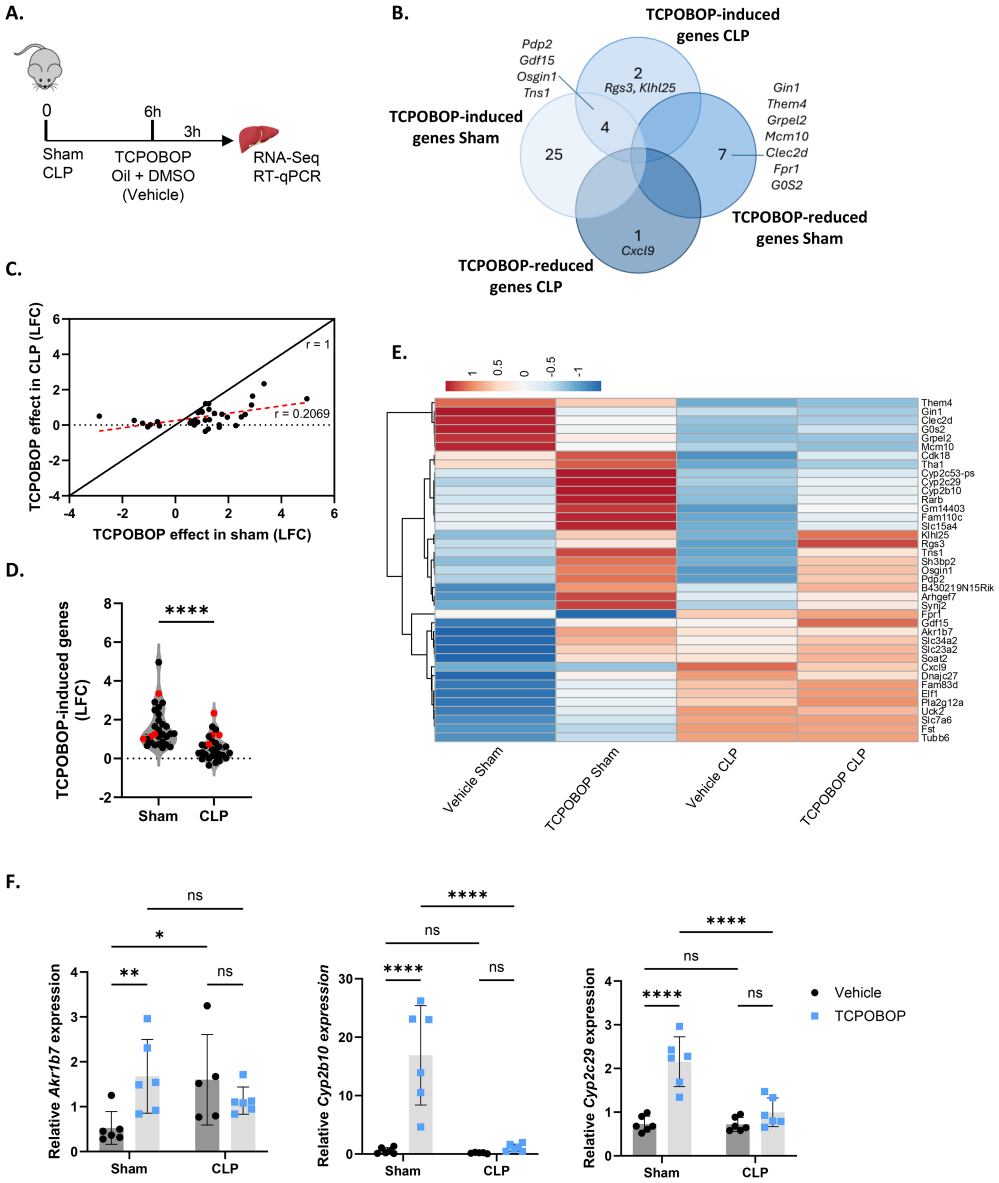
Sepsis impairs CAR transcriptional activity in the liver. **(A-F)** Mice were subjected to sham or CLP, and 6h later, TCPOBOP (12.5 mg/kg) or vehicle (corn oil + DMSO (50%/50%)) was injected intraperitoneally, followed by liver isolation 3h later for RNA-Seq and RT-qPCR analysis. P-values for differential gene expression were calculated from DESeq2 (Wald test). n=4/group (biological replicates). **(A)** Experimental setup. **(B)** Overlap between genes downregulated (LFC < 0, Padj < 0.05) by TCPOBOP in sham or CLP, and genes upregulated (LFC > 0, Padj < 0.05) by TCPOBOP in sham or CLP. **(C)** Scatter plot showing the log2 fold change (LFC) of differentially expressed genes in sham upon TCPOBOP injection (Padj < 0.05) versus their LFC in CLP upon TCPOBOP injection. An r-value of 1 represents the expected slope if no difference in the effect of TCPOBOP is observed between sham or CLP mice. The red dotted line indicates the actual slope of the data, as determined by linear regression. **(D)** Violin plot showing the LFC of genes upregulated in sham upon TCPOBOP injection (LFC > 0, Padj < 0.05), referred to as TCPOBOP-induced genes, versus their LFC in CLP upon TCPOBOP injection. (n=26/group). P-value was analyzed with unpaired t-test. **(E)** Heatmap of differentially expressed genes in sham or CLP upon TCPOBOP injection (Padj < 0.05), plotted across all conditions with normalized counts as the unit scale. Rows are centered by unit variance scaling and clustered using correlation distance and average linkage. **(F)** RT-qPCR mRNA expression of representative CAR target genes *Akr1b7*, *Cyp2b10*, and *Cyp2c29*, normalized to housekeeping genes *Rpl* and *Hprt*. n=5-6/group. Bars: mean ± SD. Each dot represents a single biological replicate. P-values were analyzed with two-way ANOVA. ns, nonsignificant, *P-value < 0.05, **P-value < 0.01, ****P-value < 0.0001.

To investigate CAR activity in sepsis on a genome-wide level, sham and CLP-treated mice were injected with CAR agonist TCPOBOP or vehicle (corn oil + DMSO), 6h post-surgery (**Figure 1A**), when CLP mice display hypothermia and significant HNF4α, PPARα and GR activity problems (7–9). According to public RNA sequencing (RNA-Seq) data (GSE40120), injection of TCPOBOP in CAR KO mice showed no significant changes in gene expression, indicating its specificity for CAR. Given that CAR is mainly expressed in the liver, we isolated the livers of these mice 3h post-injection and performed bulk RNA-Seq. Only 36 genes were responsive to TCPOBOP in sham (padj < 0.05) (**Figure 1B**). When plotting the log2 fold change (LFC) of these 36 genes in sham mice upon TCPOBOP injection versus their LFC in CLP mice with TCPOBOP, a clear reduction in response to TCPOBOP was observed in the CLP group (**Figure 1C**). On average, only 20.7% of the gene expression changes observed in sham mice upon TCPOBOP remained in CLP mice upon TCPOBOP. For the 29 genes that were upregulated by TCPOBOP in sham, their LFC was significantly less high in CLP relative to sham (**Figure 1D**). Of these 29 genes, only 4 remained significantly upregulated by TCPOBOP in CLP (highlighted in red). Additionally, 2 genes were specifically upregulated and 1 was downregulated (padj < 0.05) by TCPOBOP in CLP and not in sham (**Figure 1B**). All 39 TCPOBOP responsive genes are shown in the heatmap (**Figure 1E**): some of the genes upregulated by TCPOBOP in sham showed a trend towards downregulation in vehicle CLP, except for *Cyp2c29*, which remained unchanged, and were either not induced or less induced by TCPOBOP in CLP (group 1). Others, which were upregulated by TCPOBOP in sham, showed a trend towards upregulation in vehicle CLP but exhibited no further increase in response to TCPOBOP in CLP, except for *Gdf15* (group 2). Many of the genes in the first group are involved in hepatic metabolism. For example, *Cyp2b10*, *Cyp2c29*, and *Cyp2c53* play a role in drug metabolism; *Pdp2* mediates the dephosphorylation of pyruvate dehydrogenase, which is required for its activation and the subsequent conversion of pyruvate into acetyl-CoA; and *Rarb* regulates the transcription of genes involved in lipid metabolism. On the other hand, genes in the second group are involved in cell cycle regulation (*Fam83d*, *Tubb6*, *Uck2*), transport of ascorbic acid (*Slc23a2*), inorganic phosphate (*Slc34a2*), amino acids (*Slc7a6*), and cell communication and/or migration (*Arhgef7*, *Sh3bp2*, *Synj, Tns1*). Examples of genes that illustrate TCPOBOP resistance in CLP; we validated 3 genes by RT-qPCR (**Figure 1F**).

### HNF4α and PPARα regulate *Nr1i3* mRNA expression levels in sepsis

**Figure 2:**
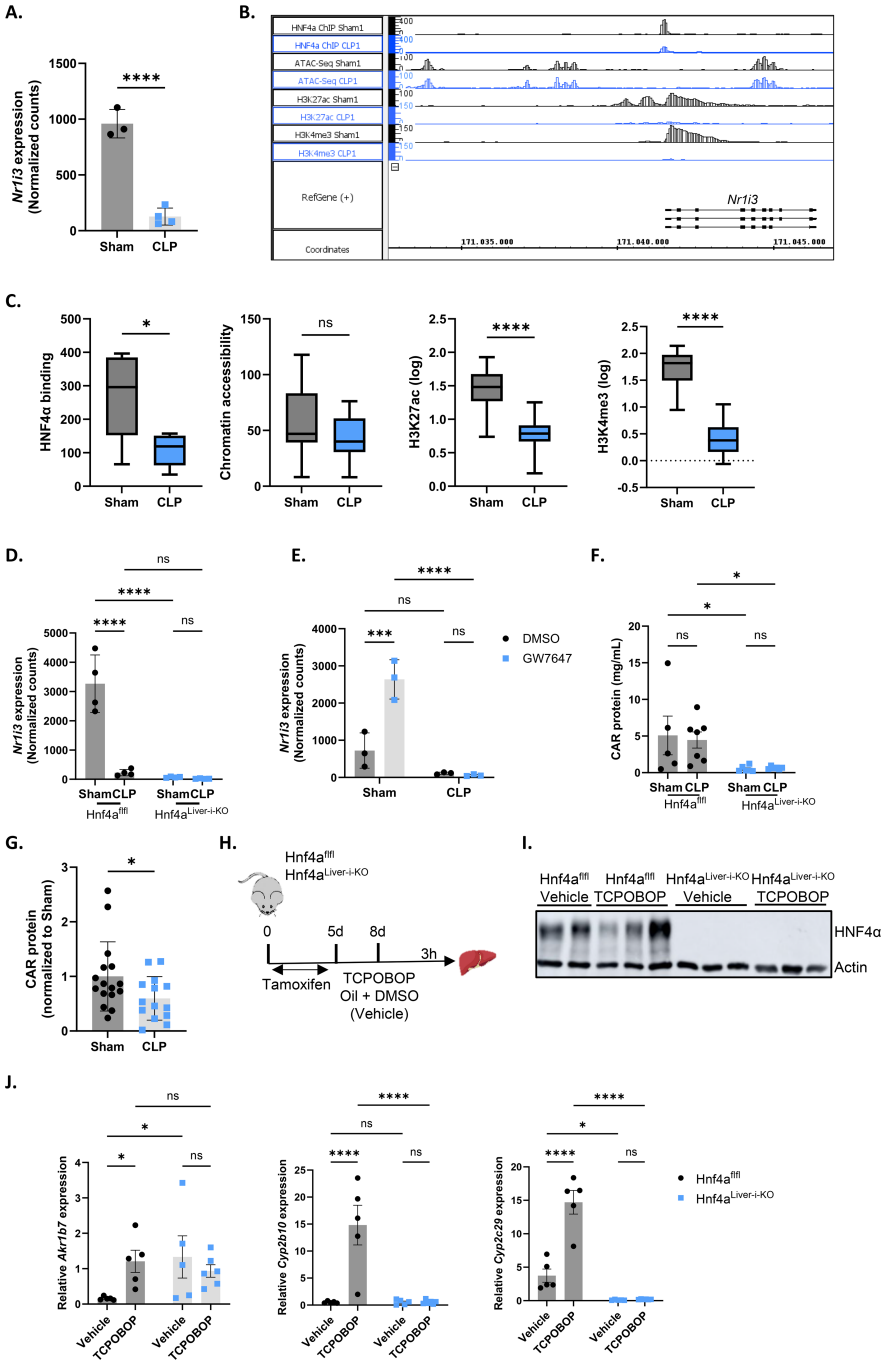
HNF4α and PPARα regulate *Nr1i3* mRNA expression levels in sepsis. **(A)** Normalized counts of *Nr1i3* in the liver from mice subjected to sham or CLP surgery, followed by intraperitoneal administration of corn oil + DMSO (50%/50%) 6h later, and liver isolation 3h thereafter (RNA-Seq data). n=4/group. **(B)** Integrated genome browser (IGB) view of representative bedgraph files showing peaks around the *Nr1i3* gene from HNF4α ChIP-Seq, ATAC-Seq, H3K27ac ChIP-Seq, and H3K4me3 ChIP-Seq data from the liver 8h after sham or CLP. The peak height represents the amount of reads mapped to genome. **(C)** Boxplots showing the area under the curve of peaks identified in panel **(B)**, plotted separately for HNF4α ChIP-Seq, ATAC-Seq, H3K27ac ChIP-Seq, and H3K4me3 ChIP-Seq. n=4/group. The boxplots display the distribution from Q1 (the first quartile, 25%) to Q3 (the third quartile, 75%), where the box itself spans the interquartile range (IQR). The line inside the box indicates the median. The whiskers extend from Q1 - 1.5 * IQR to Q3 + 1.5 * IQR. **(D)** Normalized counts of *Nr1i3* in livers from Hnf4a^Liver-i-KO^ and Hnf4a^flfl^ mice three days after tamoxifen treatment, 8h after sham or CLP surgery (RNA-Seq data). n=4/group. **(E)** Normalized counts of *Nr1i3* in livers from mice that underwent sham or CLP surgery and received an injection of GW7647 (10 µg/g) or DMSO 6h post-surgery, followed by liver isolation 4h later (RNA-Seq data). n=3/group. **(F)** CAR protein levels in the liver of Hnf4a^Liver-i-KO^ and Hnf4a^flfl^ mice three days after tamoxifen treatment and 8h after sham or CLP surgery, as measured by ELISA. n=5-7/group. **(G)** CAR protein levels in the liver of sham or CLP mice, as measured by ELISA. Samples were collected at different timepoints between 24h and 96h after CLP. n=14-16/group. **(H–J)** Hnf4a^flfl^ and Hnf4a^Liver-i-KO^ mice were injected with tamoxifen for 5 consecutive days, followed by an injection of TCPOBOP (12.5 mg/kg) or corn oil + DMSO (50%/50%) 3 days later. The liver was then isolated 3h after injection for RT-qPCR analysis. n=5-6/group. **(H)** Experimental setup. **(I)** Western blot analysis of HNF4α protein levels (54 kDa) relative to actin (42 kDa). **(J)** RT-qPCR mRNA expression of representative CAR target genes *Akr1b7*, *Cyp2b10*, and *Cyp2c29*, normalized to housekeeping genes *Rpl* and *Gapdh*. Bars: mean ± SD **(A, D, E, G)**, or SEM **(F, J)**. Each dot represents a single biological replicate. P-values were analyzed with unpaired t-test **(A, C, G)** or two-way ANOVA **(D–F, J)**. ns, nonsignificant, *P-value < 0.05, ***P-value < 0.001, ****P-value < 0.0001.

To investigate the mechanism responsible for the reduced CAR transcriptional activity during sepsis, we first examined whether *Nr1i3* mRNA expression was affected. At 9h after CLP, hepatic *Nr1i3* mRNA levels were indeed significantly decreased (**Figure 2A**). This decrease was consistent at multiple timepoints post-CLP, including 6h, 8h, 10h and 24h (**Supplementary Figure S1**). *Nr1i3* transcription is primarily regulated by HNF4α, PPARα and PGC1α (14, 55, 56). We previously reported that HNF4α progressively loses its function in the liver during sepsis, driven by reduced chromatin binding, leading to decreased H3K27 acetylation (H3K27ac) and, to a lesser extent, altered chromatin accessibility. The observed decreases in acetylation and chromatin accessibility may consequently contribute to the downregulation of various nuclear receptors, as shown for PPARα (9). We found reduced binding of HNF4α at the *Nr1i3* promoter 8h after CLP, which was associated with a decrease in H3K27ac and H3K4 methylation (H3K4me3), but not with changes in chromatin accessibility (**Figures 2B, C**). Since H3K27ac marks active enhancers, while H3K4me3 marks active promoters, the losses of these marks suggest reduced enhancer and promoter activity, which is consistent with the decreased transcription of *Nr1i3* observed in both CLP mice and mice with hepatic HNF4α depletion, three days after tamoxifen administration (Hnf4a^Liver-i-KO^) (**Figure 2D**). The PPARα agonist GW7647 upregulates *Nr1i3* mRNA expression in sham mice but not in CLP mice, indicating that both HNF4α and PPARα may contribute to the downregulation of *Nr1i3* in CLP (**Figure 2E**). This lack of response to GW7647 has been previously attributed to the downregulation of *Ppara* in the liver 6h after CLP (8). Nevertheless, CAR protein was significantly downregulated in Hnf4a^Liver-i-KO^ mice, both in sham and CLP, compared to Hnf4a^flfl^ mice, but not in Hnf4a^flfl^ mice 8h after CLP (**Figure 2F**), but was decreased at later stages of sepsis, between 24h and 96h after CLP (**Figure 2G**). Since the decline in CAR transcriptional activity in response to TCPOBOP precedes it quantitative decline at the protein level, several mechanisms impacting its activity during sepsis might be at play.

To confirm the dependency of CAR activity on HNF4α, Hnf4a^flfl^ and Hnf4a^Liver-i-KO^ mice were injected with TCPOBOP or vehicle three days after tamoxifen administration, when livers were depleted of HNF4α, and livers were harvested 3h post-injection for RT-qPCR analysis (**Figures 2H, I**). None of the CAR target genes tested showed any response to TCPOBOP in the KO, indicating fully impaired CAR activity (**Figure 2J**). However, similar to CLP, *Akr1b7* was basally upregulated in Hnf4a^Liver-i-KO^ mice. Given the loss of CAR protein in these mice, these findings suggest that transcription factors other than CAR may be responsible for *Akr1b7* upregulation in CLP as a compensatory mechanism. PXR is a strong candidate, as it shares many target genes and binding partners with CAR and also upregulates *Akr1b7* upon administration of its agonist PCN (57, 58). In conclusion, these findings suggest that the loss of HNF4α and PPARα function during sepsis disrupts *Nr1i3* transcription, thereby impairing CAR activity. However, this reduction only partially accounts for the diminished transcriptional activity of CAR during sepsis, indicating that additional mechanisms are also involved.

### HNF4α-driven chromatin remodeling contributes to impaired CAR DNA binding in sepsis

**Figure 3:**
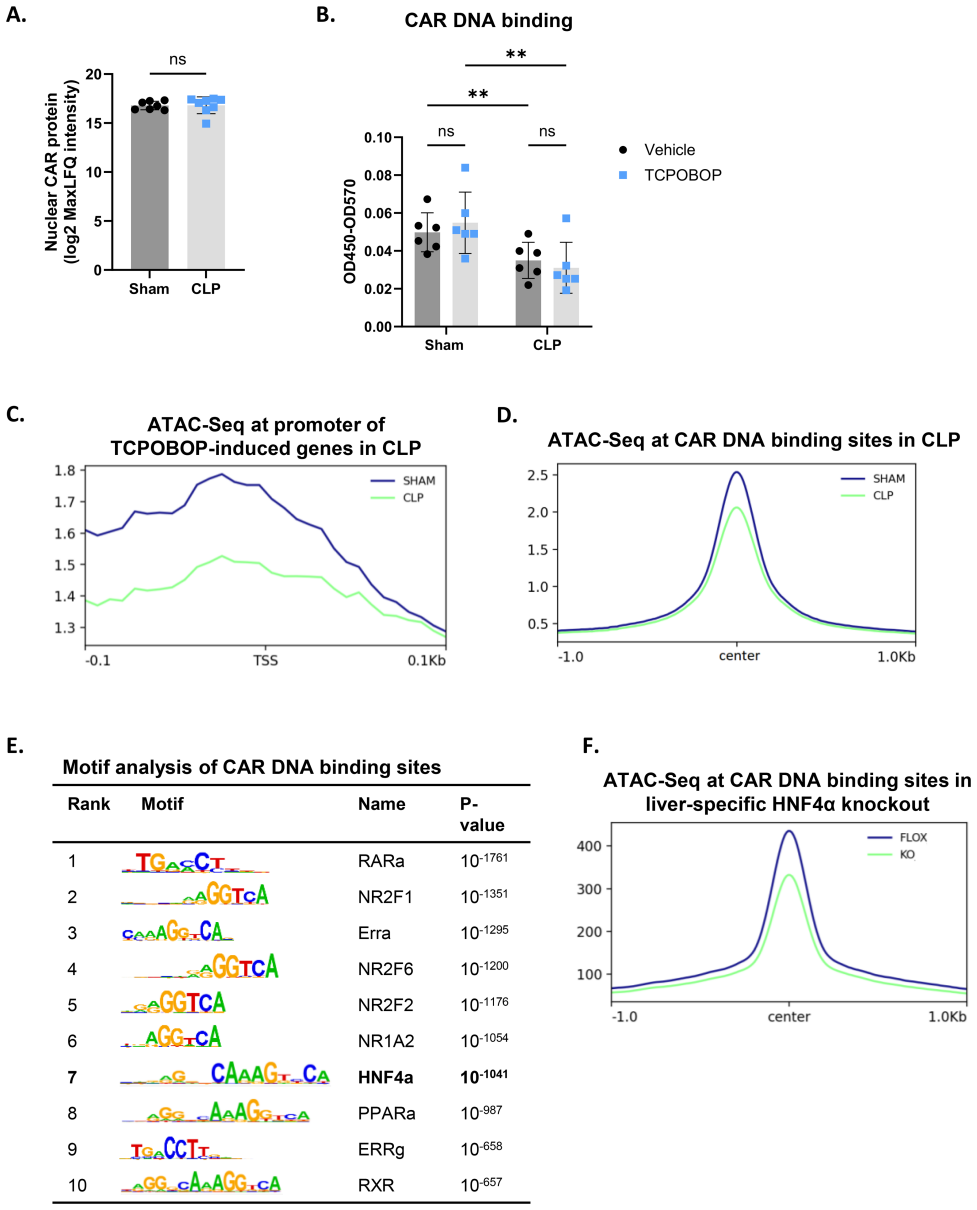
HNF4α-driven chromatin remodeling contributes to impaired CAR DNA binding in sepsis. **(A)** Liver nuclear lysates were prepared from mice 8h after sham or CLP surgery for mass spectrometry analysis. Nuclear CAR protein levels were quantified, expressed as log2 MaxLFQ Intensity. Statistical analysis was performed using the limma package, with FDR < 0.05 and |log2FC| = 1. n=7-8/group. **(B)** Mice were subjected to sham or CLP, and 6h later, TCPOBOP (12.5 mg/kg) or vehicle (corn oil + DMSO (50%/50%)) was injected intraperitoneally, followed by liver isolation 3h later. Nuclear lysates were prepared for CAR DNA binding assay. OD450-OD570 represents the strength of CAR binding to dsDNA oligos immobilized on a plate. n=6/group. **(C)** Sham and CLP ATAC signals centered at 0.1 kbp around the transcription start site (TSS) of genes upregulated (LFC > 0, Padj < 0.05) by TCPOBOP in sham, referred to as TCPOBOP-induced genes, based on the TCPOBOP RNA-Seq from **Figure 1**. P-values for were calculated using DESeq2 (Wald test). Region scores were calculated by deepTools. n=4/group. **(D)** Sham and CLP ATAC signals centered at 1 kbp around the center of CAR ChIP-Seq peaks, identifying CAR DNA binding sites. Region scores were calculated by deepTools. n=3/group. **(E)** HOMER transcription factor motif enrichment centered at 100bp around the center of CAR ChIP-Seq peaks. P-values derived from Fisher’s exact test (Hypergeometric test). n=3/group. **(F)** Hnf4a^flfl^ (FLOX-blue) and Hnf4a^Liver-i-KO^ (KO-green) ATAC signals centered at 1 kbp around the center of CAR ChIP-Seq peaks. Region scores were calculated by deepTools. n=3/group. Bars: mean ± SD. Each dot represents a single biological replicate. P-values were analyzed with unpaired t-test **(A)** or two-way ANOVA **(B)**. ns, nonsignificant, **P-value < 0.01.

Because total CAR protein levels remained unchanged 8h after CLP, we further investigated alternative mechanisms for the clearly impaired CAR functional decline, including its nuclear localization, phosphorylation status and chromatin binding. Consistent with previous reports, CAR could already be detected in the nucleus without ligand stimulation by MS analysis (**Figure 3A**). However, no differences were observed in the liver between sham and CLP at 8h. Despite its nuclear presence, phenobarbital-mediated activation of CAR is regulated by protein phosphorylation, as the PP2A-specific phosphatase inhibitor okadaic acid reduces CAR activity following phenobarbital treatment (36). To investigate CAR phosphorylation in CLP upon TCPOBOP, CAR was immunoprecipitated from livers of sham or CLP mice injected with TCPOBOP or vehicle 6h post-surgery, and isolated 3h post-injection, followed by Western blot using a general anti-phospho antibody (**Supplementary Figure S2A**). However, CAR phosphorylation was even reduced by TCPOBOP in CLP (**Supplementary Figures S2B–D**).

We then assessed the DNA binding activity of CAR. Due to the lack of suitable CAR antibodies for ChIP-Seq, we used an *in vitro* CAR DNA binding assay. In this assay, plates containing DNA with the consensus binding site of CAR were incubated with nuclear lysates prepared from liver samples collected 3h after TCPOBOP/vehicle injection (which was administered 6h after sham or CLP). CAR DNA binding was significantly decreased in CLP conditions, both basally and following TCPOBOP treatment (**Figure 3B**). Since the DNA probe remained unchanged in these studies, these data suggest that a posttranslational modification of CAR or an altered interaction may be responsible for the reduced DNA binding in sepsis. However, the chromatin region 100 bp upstream of the promoters of CAR target genes upregulated by TCPOBOP in sham (29 genes), as identified in our RNA-Seq analysis from **Figure 1**, was less accessible in CLP, based on our ATAC-Seq data from livers 8h after CLP (**Figure 3C**) (9). Moreover, we overlapped this ATAC-Seq data with public CAR ChIP-Seq data from livers of YFP-mCAR transgenic mice collected 2h after the last TCPOBOP injection (59). To validate the ChIP-Seq data, 19 of the 29 genes responsive to TCPOBOP in sham were found near a peak. From this overlap, we found that chromatin sites to which CAR can bind were less accessible in CLP (**Figure 3D**). This suggests that a more closed chromatin signature in CLP might also contribute to the decreased CAR DNA binding and, consequently, its reduced transcriptional activity. CAR DNA binding sites were enriched for the *Hnf4a* motif, indicating a potential interaction between CAR and HNF4α (**Figure 3E**). Therefore, we also overlapped the CAR ChIP-Seq data with public ATAC-Seq data from livers of Hnf4a^Liver-i-KO^ mice (13) and found that CAR DNA binding sites were also less accessible in the KO, suggesting that the presence of HNF4α is crucial for maintaining an accessible chromatin state at CAR enhancers (**Figure 3F**). Consequently, these findings indicate that the loss of HNF4α function during sepsis not only decreases *Nr1i3* mRNA expression, but also reduces chromatin accessibility at CAR binding sites, thereby contributing to its impaired DNA binding in CLP.

### CAR loss-of-function disrupts hepatic metabolism but induces a beneficial hepatic acute phase response in sepsis

**Figure 4:**
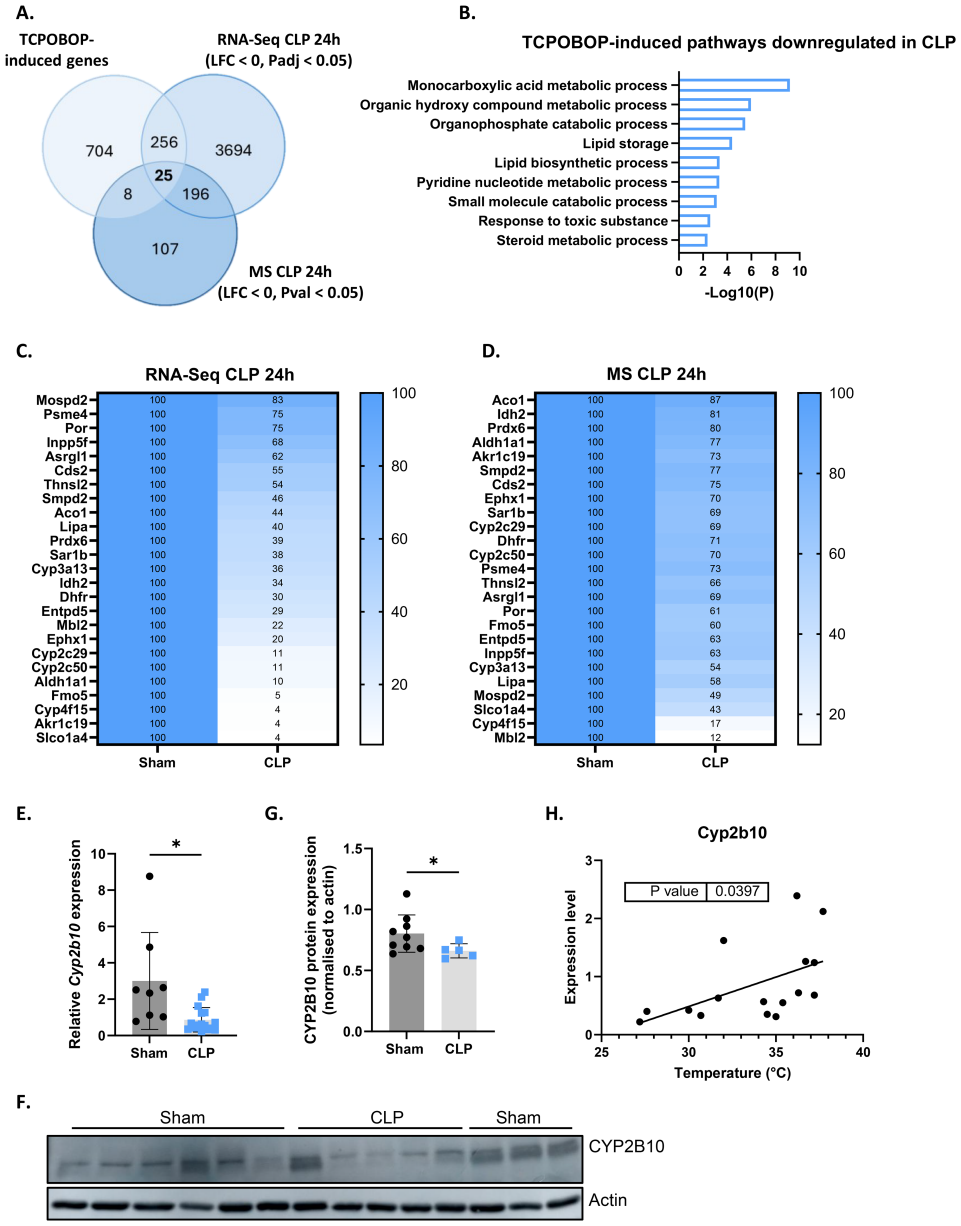
CAR loss-of-function disrupts hepatic metabolism in sepsis. **(A)** Overlap between genes upregulated (LFC > 0, Padj < 0.05) by TCPOBOP in non-operated wild-type mice that received 4 injections of TCPOBOP (3 mg/kg) or corn oil + DMSO (90%/10%) -referred to as TCPOBOP-induced genes- derived from public RNA-Seq data, and genes downregulated at the RNA (RNA-Seq; LFC < 0, Padj < 0.05) and protein (MS; LFC < 0, Pval < 0.05) level in the liver 24h after CLP. For MS, n=4/group. Statistical analysis was performed using the limma package, with FDR < 0.05 and |log2FC| = 1. For RNA-Seq: n=3/group. P-values were calculated using DESeq2 (Wald test). **(B)** Metascape pathway analysis of the genes from the overlap in panel **(A)**, referred to as TCPOBOP-induced pathways downregulated in CLP. P-values derived from Fisher’s exact test (Hypergeometric test). **(C, D)** Heatmaps of the expression levels of genes **(C)** or proteins **(D)** from the overlap in panel **(A)**, with expression plotted relative to sham (set at 100%), and the genes or proteins ordered by their percentage in CLP from highest to lowest. **(E)** RT-qPCR analysis of *Cyp2b10* mRNA expression, normalized to housekeeping genes *Gapdh*, *Rpl* and *Hprt*, in the liver of mice 24h after sham or CLP surgery. n=8-16/group. **(F, G)** Western blot analysis of CYP2B10 (58 kDa) relative to actin (40 kDa) in the liver of mice 24h after sham or CLP surgery. n=5-9/group. **(H)** Correlation between body temperature and *Cyp2b10* mRNA expression, measured by RT-qPCR and normalized to housekeeping genes *Gapdh*, *Rpl* and *Hprt*, in the liver of mice 24h after CLP surgery. P-values were calculated by linear regression. Bars: mean ± SD. Each dot represents a single biological replicate. For E and G, p-values were analyzed with unpaired t-test. *P-value < 0.05.

**Figure 5:**
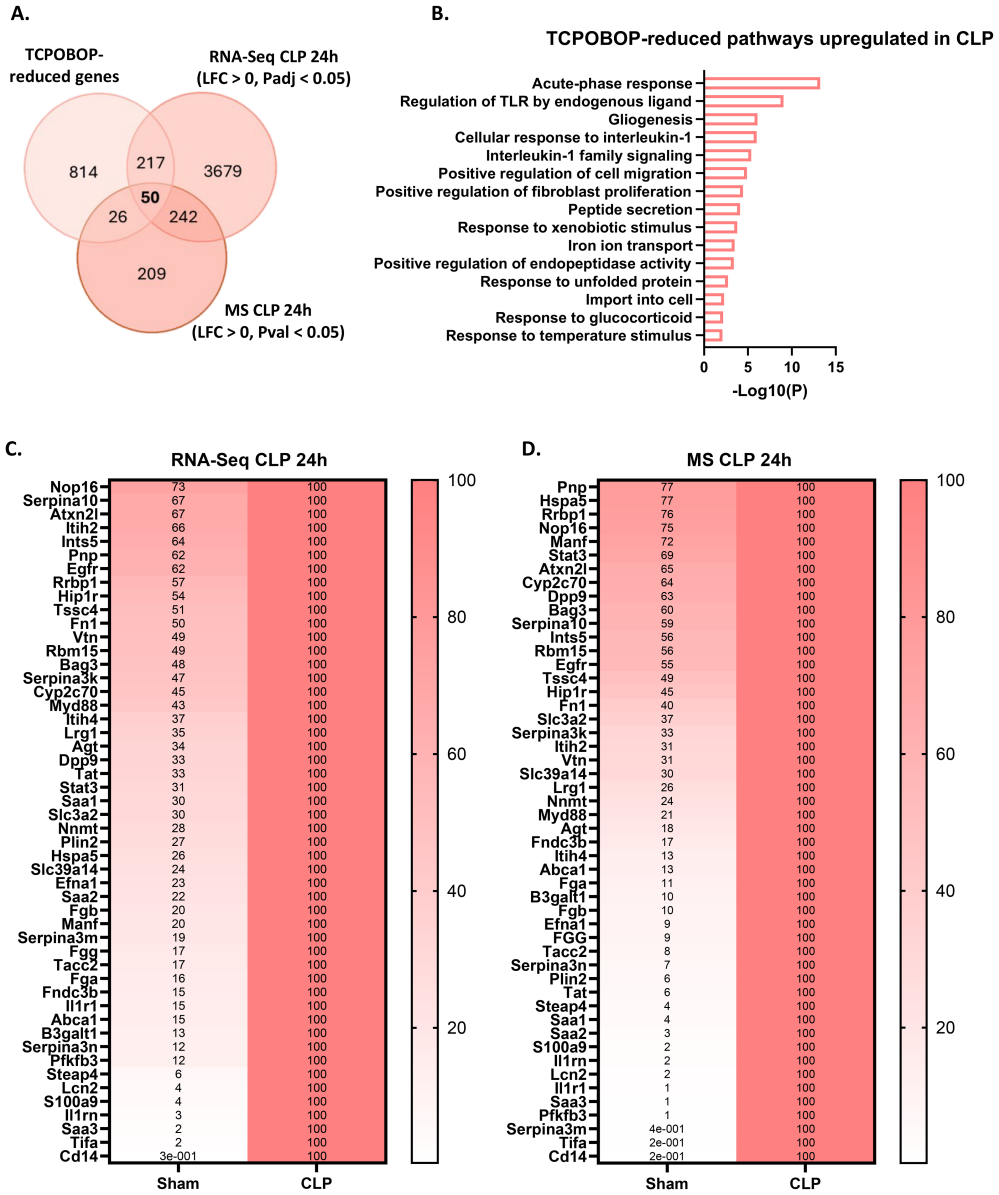
CAR loss-of-function induces a beneficial hepatic acute phase response in sepsis. **(A)** Overlap between genes downregulated (LFC < 0, Padj < 0.05) by TCPOBOP in non-operated wild-type mice that received 4 injections of TCPOBOP (3 mg/kg) or corn oil + DMSO (90%/10%) -referred to as TCPOBOP-reduced genes- derived from public RNA-Seq data, and genes upregulated at the RNA (RNA-Seq; LFC > 0, Padj < 0.05) and protein (MS; LFC > 0, Pval < 0.05) level in the liver 24h after CLP. For MS, n=4/group. Statistical analysis was performed using the limma package, with FDR < 0.05 and |log2FC| = 1. For RNA-Seq: n=3/group. P-values were calculated using DESeq2 (Wald test). **(B)** Metascape pathway analysis of the genes from the overlap in panel **(A)**, referred to as TCPOBOP-reduced pathways upregulated in CLP. P-values derived from Fisher’s exact test (Hypergeometric test). **(C, D)** Heatmaps of the expression levels of genes **(C)** or proteins **(D)** from the overlap in panel **(A)**, with expression plotted relative to CLP (set at 100%), and the genes or proteins ordered by their percentage in Sham from highest to lowest.

The CAR target genes identified in our RNA-Seq analysis provide initial insights into the biological processes affected by CAR during sepsis. However, due to the limited transcriptional response to a single TCPOBOP injection in our experiments, we expanded our analysis to other CAR dependent genes that have been identified by other researchers and are available as public RNA-Seq data (58). These authors identified 2100 CAR dependent genes (993 induced and 1107 reduced) based on repetitive TCPOBOP injections. Their approach, involving naive mice and multiple injections, yielded a stronger transcriptional response in the liver. We focused on their 993 TCPOBOP-induced (CAR dependent) genes and overlapped these with genes downregulated in the liver 24h after CLP at both the RNA and protein level (**Figure 4A**). The genes were identified using public RNA-Seq data 24h after sham or CLP (7), and the proteins were identified through MS proteomics on livers collected at the same timepoint. This timepoint was chosen because metabolic changes during sepsis are most pronounced at this stage, characterized by hypothermia, hypoglycemia, hyperlactatemia, and hepatic steatosis (7, 8). The 25 genes/proteins within the overlap (**Figure 4A**) are primarily involved in the metabolism of monocarboxylic acids (**Figure 4B**). These 25 are listed as mRNAs and proteins in **Figures 4C, D**, respectively, with their levels normalized to 100% in the sham mice. For example, *Cyp2c29* and *Cyp2c50* catalyze the epoxidation of arachidonic acid, *Ephx1* facilitates the hydrolysis of epoxyeicosanoids, *Aldh1a1* is involved in the metabolism of retinoic acid, and *Lipa* catalyzes the hydrolysis of triglycerides. Furthermore, *Cyp4f15* participates in the catabolism of leukotriene B4, *Thnsl2* contributes to the biosynthesis of 2-oxobutyrate, *Akr1c19* plays a role in the metabolism of jasmonic acid, and *Fmo5* contributes to the oxidation of short-chain fatty acids. Among the 25 genes, *Slco1a4*, *Akr1c19*, and *Cyp4f15* are the most downregulated at the RNA level, with *Slco1a4* being involved in the transport of bile acids and other monocarboxylic acids (**Figure 4C**). On the other hand, MBL2, CYP4F15, and SLCO1A4 are most strongly downregulated at the protein level, with MBL2 mediating complement activation (**Figure 4D**). We also identified TCPOBOP-reduced genes -genes that are significantly downregulated by TCPOBOP in the liver (1107 in total)- and overlapped them with genes upregulated in the liver 24h after CLP at both the RNA and protein level (**Figure 5A**). These 50 genes are primarily involved in the APR, including *Stat3*, *Fn1*, *Itih4*, *Ilr1n*, *Cd14*, *Agt*, *Myd88*, *S100a9*, *Egfr*, *Dpp9*, *Saa1*, *Saa2*, *Saa3*, *Il1r1*, *Serpina3m*, *Serpina3n* (**Figure 5B**). Among the 50 genes within the overlap, *Cd14*, *Tifa*, and *Saa3* are most strongly upregulated at the RNA level, with *Cd14* being mainly expressed by macrophages and interacting with LPS, while *Tifa* contributes to the activation of NFκB and MAPK signaling during inflammation (**Figure 5C**). On the other hand, CD14, TIFA, and SERPINA3M are most strongly upregulated at the protein level (**Figure 5D**). To conclude, CAR loss-of-function in sepsis disrupts hepatic metabolism, particularly monocarboxylic acid metabolism, while inducing the hepatic APR. Acute phase proteins (APPs) have anti-inflammatory and tissue repairing activities and are therefore essential in hepatic regeneration. In that regard, CAR loss-of-function in sepsis can be considered a way to promote the APR at the expense of certain liver metabolic functions.

*Cyp2b10* is a key biomarker of CAR signaling due to the presence of a phenobarbital-responsive enhancer module within its promoter, which makes it highly responsive to TCPOBOP induction (60). It plays a role in drug and lipid metabolism in the liver and is downregulated 24h after CLP at the RNA level (**Figure 4E**). However, since CYP2B10 could not be detected by MS, we verified its protein levels 24h after CLP using Western blot analysis, which confirmed its downregulation (**Figures 4F, G**). *Cyp2b10* mRNA expression also correlated with body temperature in mice subjected to CLP, suggesting that it could be a potential biomarker in sepsis (**Figure 4H**).

### The CAR inhibitor CINPA1 worsens sepsis survival, associated with disrupted hepatic metabolism, systemic inflammation and organ damage

**Figure 6:**
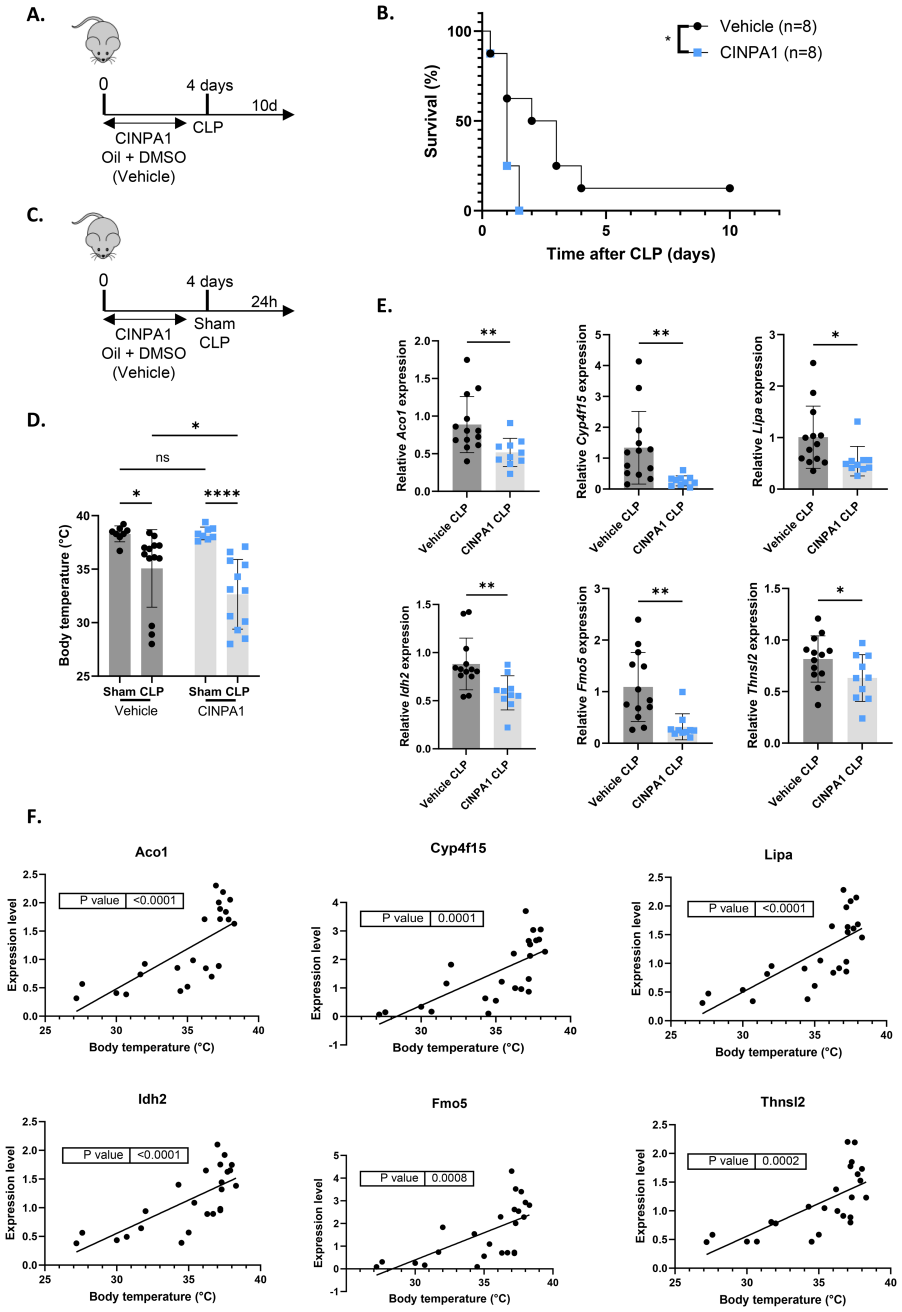
The CAR inhibitor CINPA1 worsens sepsis survival, associated with disrupted hepatic metabolism. **(A, B)** Mice were administered CINPA1 (12.5 mg/kg) or corn oil + DMSO (90%/10%) intraperitoneally for 4 days, with CLP initiated on day 4, and mortality monitored over 10 days. Data were analyzed with Log-rank test. n=8/group. **(C-F)** Mice were injected intraperitoneally with CINPA1 (12.5 mg/kg) or corn oil + DMSO (90%/10%) for 4 days, subjected to sham or CLP surgery on day 4, and blood and liver were isolated 24h later. n=8-13/group. **(C)** Experimental setup. **(D)** Body temperature. **(E)** RT-qPCR mRNA expression of representative CAR target genes *Aco1*, *Cyp4f15*, *Lipa*, *Idh2*, *Fmo5* and *Thnsl2* from the overlap in **Figure 4A**, normalized to housekeeping genes *Rpl* and *Hprt*. **(F)** Correlation between body temperature and expression levels of representative CAR target genes *Aco1*, *Cyp4f15*, *Lipa*, *Idh2*, *Fmo5* and *Thnsl2* from the overlap in **Figure 4A**, measured by RT-qPCR and normalized to housekeeping genes *Gapdh*, *Rpl* and *Hprt*, using liver samples from untreated wild-type mice collected 24h after sham or CLP. P-values were calculated by linear regression. Bars: mean ± SD. Each dot represents a single biological replicate. P-values were analyzed with unpaired t-test **(E)** or two-way ANOVA **(D)**. ns, nonsignificant, *P-value < 0.05, **P-value < 0.01, ****P-value < 0.0001.

To further investigate the functional consequences of hepatic CAR loss-of-function in sepsis, mice were treated for 4 days with the CAR inhibitor CINPA1 or with vehicle (**Figure 6A**). Importantly, qPCR analysis of several CAR target genes, selected from the 993 TCPOBOP-induced genes from **Figure 4**, showed that CINPA1 functions as only a partial inhibitor (**Supplementary Figure S3**). As mentioned earlier, this might be explained by the fact that CAR and PXR share many binding partners and target genes, suggesting that they could compensate for each other’s activities upon inhibition, as reflected by the upregulation of several CAR target genes upon CLP or HNF4α depletion in the liver (57, 58). However, such compensation has not been described in the literature yet. Nevertheless, CINPA1 treatment significantly increased mortality in CLP compared to vehicle treatment, indicating that CAR plays a crucial role in the response to sepsis (**Figure 6B**). To further assess the reduced survival associated with CINPA1, liver and blood samples were collected 24h after sham or CLP surgery (**Figure 6C**). This timepoint effectively reflects the increased lethality of CINPA1 in CLP, as the body temperature was already further decreased (**Figure 6D**). Of the 25 TCPOBOP-induced genes previously shown to be downregulated 24h after CLP compared to sham, at both the RNA and protein level (**Figure 4A**), 6 representative genes were selected and showed further suppression by CINPA1 in CLP (**Figure 6E**). Using samples from untreated sham and CLP mice, we found that the expression of these genes correlated with body temperature at 24h after CLP (**Figure 6F**). As mentioned earlier, many of these genes are involved in monocarboxylic acid metabolism, except for *Aco1* and *Idh2*, which play roles in the TCA cycle by catalyzing the isomerization of citrate to isocitrate and the oxidative decarboxylation of isocitrate to α-ketoglutarate, respectively.

**Figure 7:**
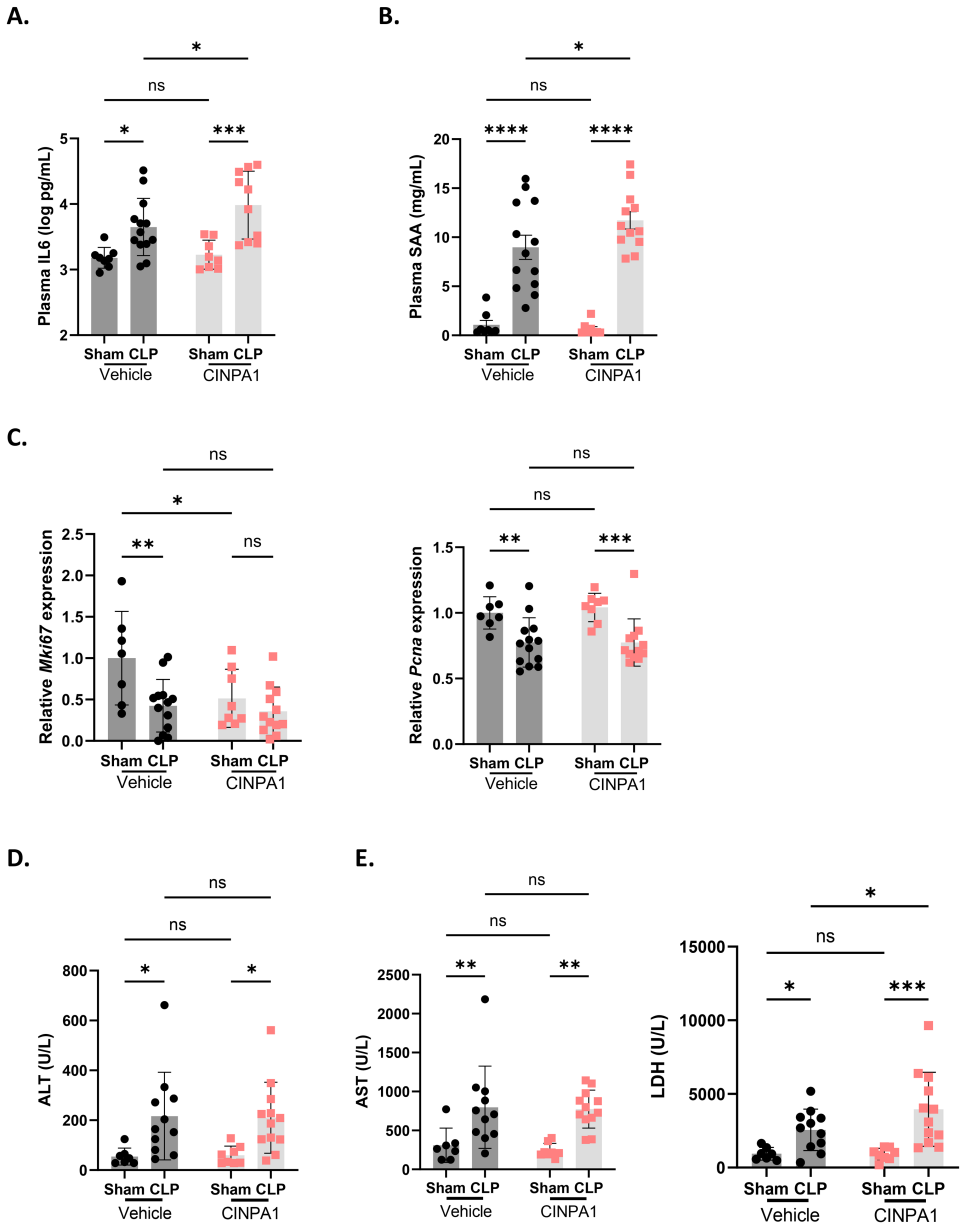
The CAR inhibitor CINPA1 worsens sepsis survival, associated with systemic inflammation, an elevated hepatic acute phase response and increased organ damage. **(A-E)** Mice were injected intraperitoneally with CINPA1 (12.5 mg/kg) or corn oil + DMSO (90%/10%) for 4 days, subjected to sham or CLP surgery on day 4, and blood and liver were isolated 24h later. n=8-13/group. **(A, B)** IL6 and SAA protein levels in the plasma, as measured by ELISA. **(C)** RT-qPCR mRNA expression of proliferation markers *Mki67* and *Pcna*, normalized to housekeeping genes *Hprt* and *Rpl*. **(D, E)** Plasma alanine aminotransferase (ALT), aspartate aminotransferase (AST) and lactate dehydrogenase (LDH) levels. Bars: mean ± SD **(A, C–E)** or SEM **(B)**. Each dot represents a single biological replicate. P-values were analyzed with two-way ANOVA. ns, nonsignificant, *P-value < 0.05, **P-value < 0.01, ***P-value < 0.001, ****P-value < 0.0001.

IL6 is a proinflammatory cytokine and a marker of systemic inflammation, with an additional role in the priming phase of liver regeneration. Following partial hepatectomy, IL6 is rapidly produced by Kupffer cells, endothelial cells and hepatocytes, inducing APP production and stimulating hepatocyte proliferation (61, 62). IL6-deficient mice show impaired liver regeneration, characterized by liver necrosis and failure (63). Plasma IL6 levels were further increased with CINPA1 in CLP, suggesting enhanced systemic inflammation upon CAR inhibition and supporting a role for CAR in suppressing immune activation (**Figure 7A**). Consistent with this increase, plasma SAA protein levels were further elevated by CINPA1 in CLP (**Figure 7B**). SAA is one of the best validated APPs in mice. This result further confirms the involvement of CAR in suppressing the hepatic APR and suggests that its loss-of-function during sepsis contributes to APR induction, possibly indirectly by enhancing plasma IL6 levels. Although APPs promote liver regeneration by supporting tissue repair and limiting infection, CAR also regulates hepatocyte proliferation, with several proliferation markers upregulated following its activation by TCPOBOP. Consequently, CAR KO mice show increased mortality after 2/3^th^ partial hepatectomy due to impaired hepatocyte proliferation (20). The proliferation markers *Mki67* and *Pcna* remained downregulated in CLP mice treated with CINPA1, with *Mki67* already suppressed in sham mice upon CAR inhibition (**Figure 7C**). Furthermore, plasma alanine aminotransferase (ALT) levels, a biomarker of impaired liver function and hepatocellular injury (64), remained upregulated in CLP mice following CINPA1 treatment (**Figure 7D**). Both features suggest that, although APPs are upregulated following CAR inhibition in CLP, liver regeneration may not be improved. CINPA1 also led to increased organ damage in CLP, as indicated by elevated lactate dehydrogenase (LDH) levels in the blood (**Figure 7E**). However, ALT and aspartate aminotransferase (AST) levels were not further increased by CINPA1, suggesting that the increased damage is not specifically related to the liver, heart, or muscle, but is more generalized. To summarize, CINPA1 worsens sepsis survival, associated with the downregulation of genes involved in monocarboxylic acid metabolism, lipid metabolism, and the TCA cycle; increased systemic inflammation; and greater organ damage. Although the hepatic APR was further upregulated, no additional signs of improved liver regeneration were observed.

**Figure 8:**
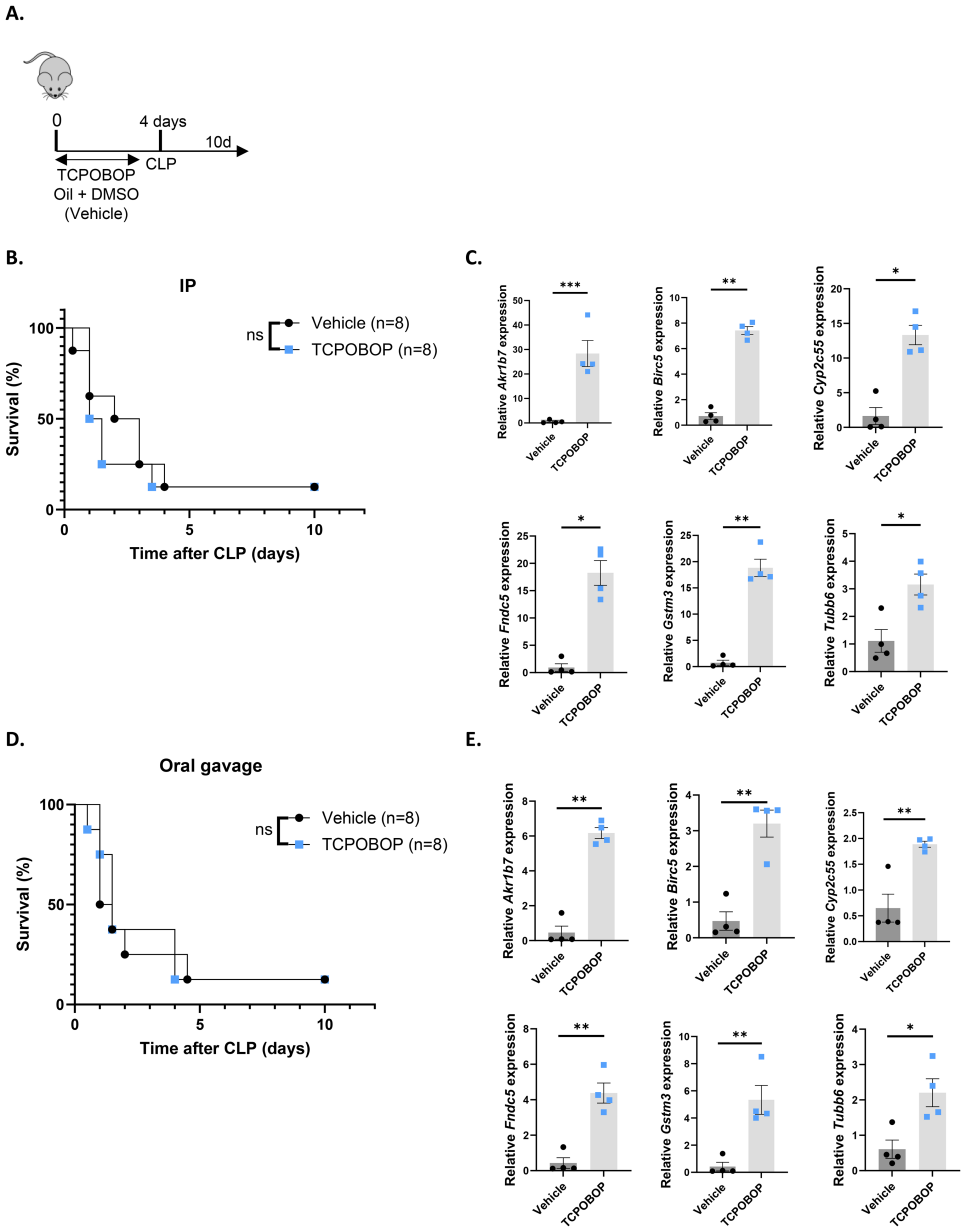
The CAR agonist TCPOBOP does not protect in sepsis. **(A, B, D)** Mice were administered TCPOBOP (3 mg/kg) or corn oil + DMSO (90%/10%) intraperitoneally (IP) or via oral gavage for 4 days, with CLP initiated on day 4, and mortality monitored over 10 days. Data were analyzed with Log-rank test. n=8/group. **(C, E)** Mice were administered TCPOBOP (3 mg/kg) or corn oil + DMSO (90%/10%) IP **(C)** or via oral gavage **(E)** for 4 days, with livers isolated on day 4 for RT-qPCR analysis of representative CAR target genes *Akr1b7*, *Birc5*, *Tubb6*, *Cyp2c55*, *Fndc5*, and *Gstm3*, normalized to housekeeping genes *Rpl* and *Hprt*. These genes were identified from public RNA-Seq data from mouse livers following 4 TCPOBOP injections. n=4/group. Bars: mean ± SEM. Each dot represents a single biological replicate. P-values were analyzed with unpaired t-test. *P-value < 0.05, **P-value < 0.01, ***P-value < 0.001.

Since inhibiting endogenous CAR activity with CINPA1 sensitized mice to sepsis, we aimed to determine whether CAR activation by TCPOBOP could provide protection. Because CAR is unresponsive to TCPOBOP post-CLP (**Figure 1**), mice were administered the CAR agonist TCPOBOP or vehicle for 4 days prior to CLP onset, with the final dose given on the day of CLP. Mortality was monitored for 10 days thereafter (**Figure 8A**). However, TCPOBOP did not improve sepsis survival, either when administered intraperitoneally (**Figure 8B**) or via oral gavage (**Figure 8D**), despite a clear increase in CAR target genes with both methods (**Figures 8C**, **E**). Taken together, our data suggest that CAR contributes to the host response during sepsis. Although ligand-mediated activation does not result in protection, the increased sensitivity observed upon CAR inhibition underscores its physiological significance.

### The HNF4α agonist NCT improves *Nr1i3* transcription and CAR activity

**Figure 9:**
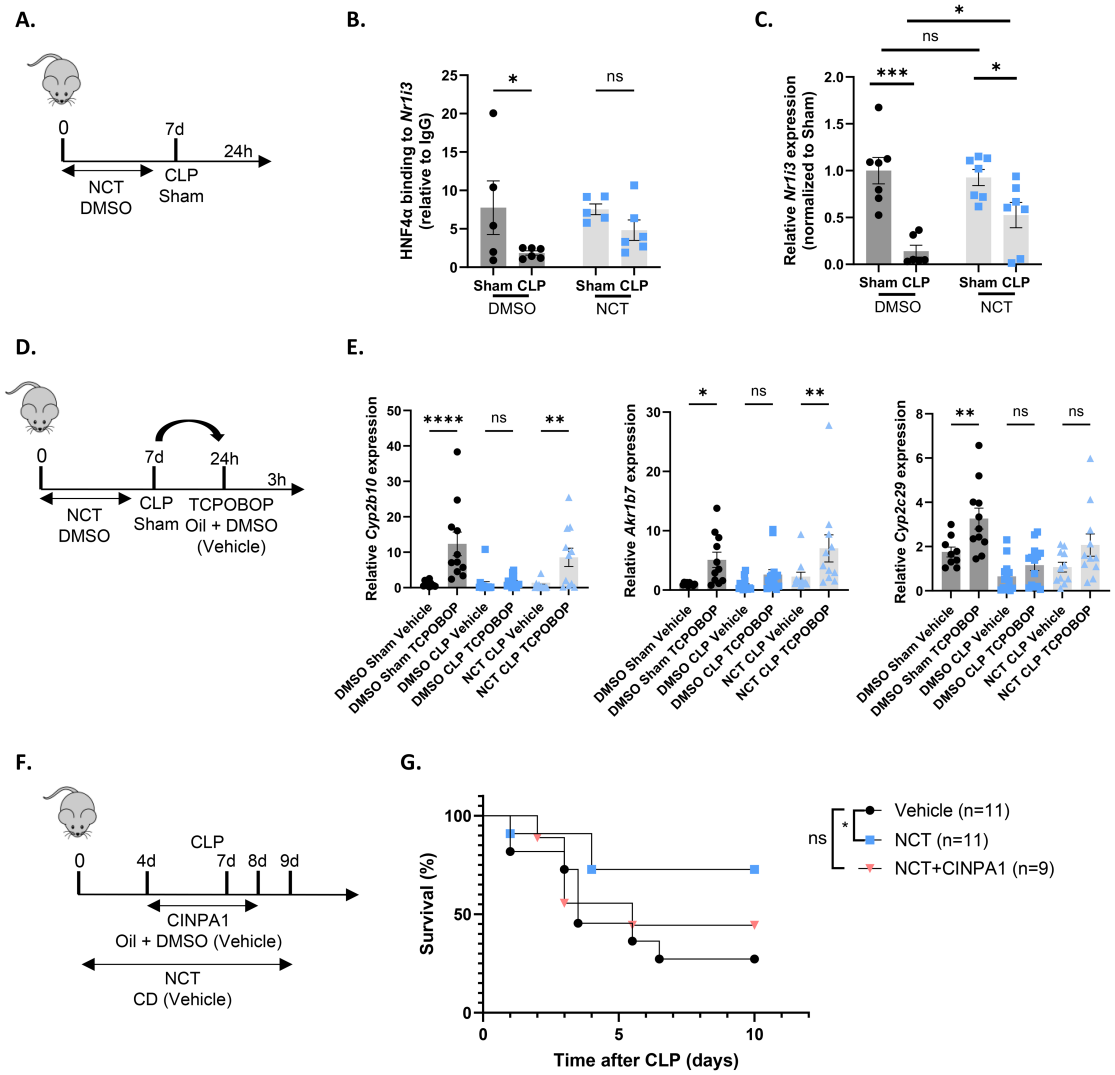
The HNF4α agonist NCT improves *Nr1i3* transcription and CAR activity. **(A–C)** Mice were injected intraperitoneally with NCT (200 mg/kg) or DMSO for 7 days: once daily for the first 3 days, twice daily for the next 3 days, and once on day 7. On day 7, they underwent either sham or CLP surgery, and blood and liver samples were collected 24h later. n=5-7/group. **(A)** Experimental setup. **(B)** HNF4α ChIP-qPCR analysis of the *Nr1i3* promoter in the liver, normalized to mouse IgG. **(C)** RT-qPCR mRNA expression of *Nr1i3*, normalized to housekeeping genes *Rpl* and *Hprt*. **(D, E)** Mice were injected intraperitoneally with NCT (200 mg/kg) or DMSO for 7 days: once daily for the first 3 days, twice daily for the next 3 days, and once on day 7. On day 7, they underwent either sham or CLP surgery, and 24h later, TCPOBOP (12.5 mg/kg) or oil + DMSO (50%/50%) was injected intraperitoneally, followed by blood and liver isolation 3h later. n=10-15/group. **(D)** Experimental setup. **(E)** RT-qPCR mRNA expression of representative CAR target genes *Akr1b7*, *Cyp2b10*, and *Cyp2c29*, normalized to housekeeping genes *Rpl* and *Hprt*. **(F, G)** Mice were injected intraperitoneally with NCT (200 mg/kg) 6 days prior to CLP, once during the first 3 days and twice during the last 3 days. On day 7, they were subjected to CLP, and NCT was administered again on the day of CLP and during the first 2 days afterward. NCT-treated mice were administered either vehicle (corn oil with 10% DMSO) or CINPA1 (12.5 mg/kg) once daily for 5 days, with 3 injections prior to CLP, one on the day of CLP and one on the following day. Control mice received both the vehicle solution for NCT (PBS with 2-hydroxypropyl-β-cyclodextrin (CD)) and the vehicle solution for CINPA1 (corn oil with 10% DMSO). Mortality was monitored for 10 days after CLP. Data were analyzed with Log-rank test. n=9-11/group. Bars: mean ± SEM. Each dot represents a single biological replicate. P-values were analyzed with one-way ANOVA **(E)** or two-way ANOVA **(B, C)**. ns, nonsignificant, *P-value < 0.05, **P-value < 0.01, ***P-value < 0.001, ****P-value < 0.0001.

NCT has previously been shown to specifically target HNF4α and provide protection in models of NAFLD and peritoneal sepsis (9, 65, 66). When NCT was administered for 7 days prior to CLP, including a final injection at the day of CLP, HNF4α chromatin binding was enhanced at target genes *Hes6*, *Apoa2*, *Ugt2b1* and *Ppara*. NCT further increased hepatic *Ppara* expression, reduced plasma FFA levels and hepatic lipid accumulation, decreased systemic inflammation and improved the hepatic APR in CLP (9). To investigate whether NCT also improves *Nr1i3* transcription in sepsis, mice were administered NCT or DMSO for 7 days, subjected to sham or CLP surgery, and the liver was isolated 24h thereafter (**Figure 9A**). NCT prevented the reduction of HNF4α binding to the *Nr1i3* promoter following sepsis, as assessed by ChIP-qPCR, thereby enhancing its transcription in the liver during CLP (**Figures 9B, C**). We also evaluated CAR activity by injecting TCPOBOP or vehicle 24h post-surgery into mice that had received NCT or DMSO for 7 days and isolated the liver 3h thereafter (**Figure 9D**). CAR activation by TCPOBOP was clearly impaired 24h after CLP, as observed earlier at the 6h timepoint, and was improved by NCT (**Figure 9E**). From this, we conclude that NCT increases both *Nr1i3* mRNA expression and CAR transcriptional activity in the liver during sepsis. To investigate whether NCT-mediated protection in CLP is dependent on CAR, mice were co-treated with NCT and CINPA1. NCT was administered daily for 7 days prior to CLP, including a final injection on the day of surgery and again 2 days post-CLP (**Figure 9F**). CINPA1 was given for 4 days prior to CLP, with the fourth injection on the day of surgery and an additional injection 1 day post-CLP. The protective effect of NCT was partially lost by CAR inhibition, indicating that CAR contributes to its protective role in CLP (**Figure 9G**).

### CAR loss-of-function in porcine sepsis and in septic mice with humanized liver

**Figure 10:**
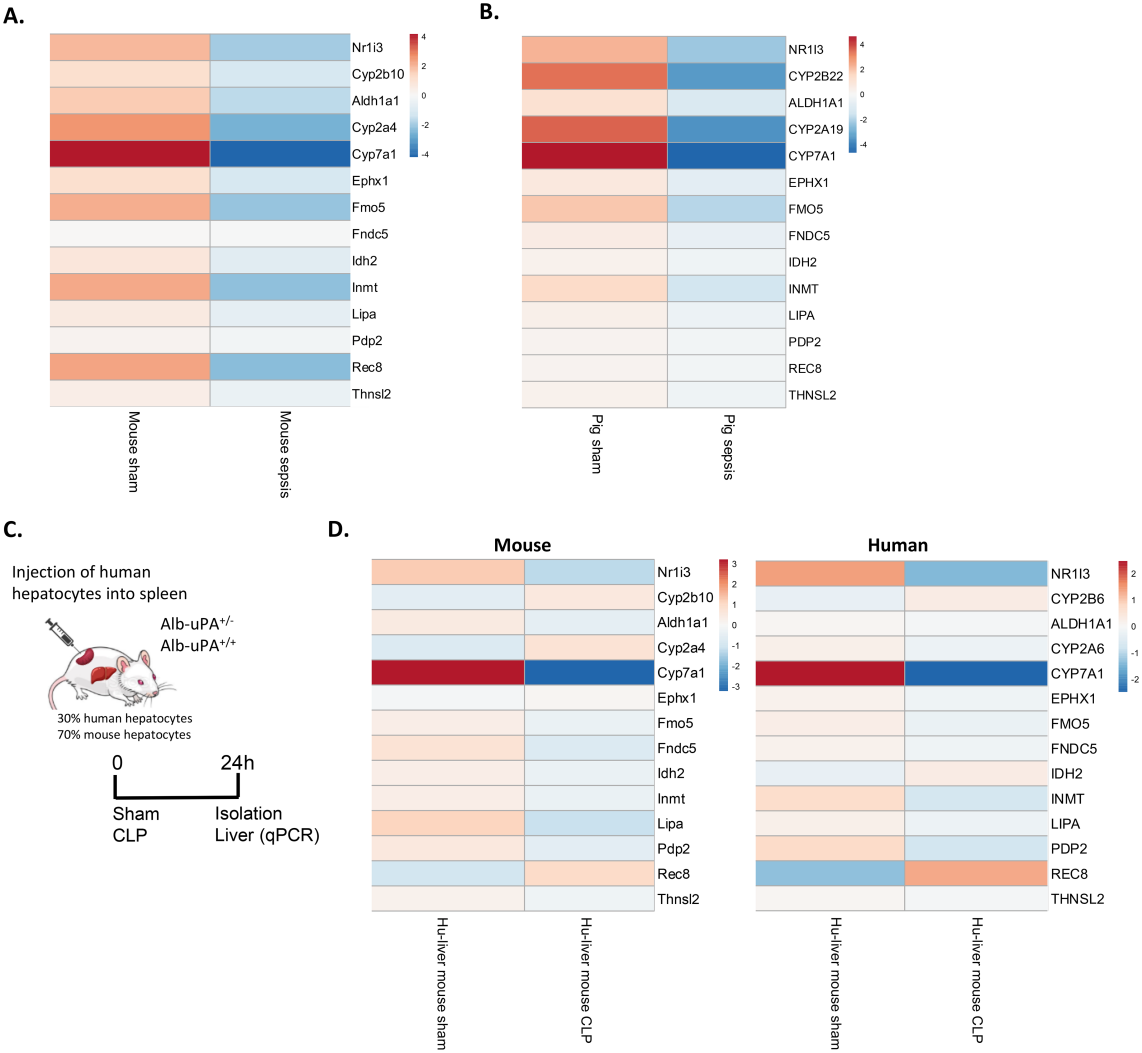
CAR loss-of-function in porcine sepsis and in septic mice with humanized liver. **(A, B)** Heatmaps showing the expression levels of *Nr1i3* and representative CAR target genes in mouse liver 24h after CLP and in pig liver 18h after intraperitoneal administration of 3g/kg autologous feces. Data are derived from RNA-Seq. Rows are centered, and the unit scale bar represents log2-normalized counts. **(C)** At two weeks, Alb-uPA^+/+^-SCID mice were injected with human hepatocytes into their spleens, enabling migration to the liver and repopulation of empty niches. After several weeks, mice underwent sham or CLP surgery, and livers were isolated 24h later. **(D)** Heatmaps showing the expression levels of *Nr1i3* and representative CAR target genes in mouse and human hepatocytes derived from humanized-liver mice 24h after sham or CLP. Expression levels were quantified by RT-qPCR relative to *Hprt* and *Rpl* (for mouse hepatocytes) and *CYPB* and *UBC* (for human hepatocytes). Human-specific and mouse-specific primers were used. Rows are centered, and the unit scale bar represents log2-expression levels.

In this study, both *Nr1i3* mRNA expression and CAR transcriptional activity were found to be reduced in the liver 6h and 24h after CLP in mice. To translate these findings, we analyzed the expression of *Nr1i3* and several CAR target genes, identified either in this study or in the literature, in RNA-Seq data from septic pigs and by qPCR in livers of humanized-liver mice 24h after sham or CLP. Septic shock in pigs was induced by intraperitoneal administration of autologous feces, resulting in fecal peritonitis. Liver samples were collected 18h after sepsis onset for bulk RNA-Seq (67). Like in mouse sepsis, *Nr1i3* and all CAR target genes investigated were downregulated in pig sepsis, although the downregulation was generally less pronounced in pigs than in mice, except for *NR1I3* and the CYP genes (**Figures 10A, B**). To generate the humanized-liver mice, severe combined immunodeficiency mice carrying the Alb-uPA transgene (uPA^+/+^) were engrafted with human hepatocytes to replace the killed mouse hepatocytes (**Figure 10C**) (43). The repopulation of human hepatocytes in the liver was determined by the ratio of human to mouse albumin in the blood of the mice, which was 30% in our case. The 70% mouse hepatocytes express the uPA transgene but derive from constant proliferation and progenitor cell differentiation. Also in these mice, *NR1I3* and the majority of CAR target genes were downregulated in CLP, in both mouse and human hepatocytes (**Figure 10D**).

## Discussion

CAR is a xenobiotic-sensing nuclear receptor that regulates the expression of drug-metabolizing enzymes, including CYP enzymes and glutathione-S-transferase, as well as transporters involved in drug metabolism, bile acid metabolism and bilirubin clearance (15). CAR has been implicated in other diseases, including alcoholic liver disease, fatty liver disease, diabetes, and cholestasis. In alcoholic liver disease, while *Nr1i3* transcription remains unaffected, both basal and phenobarbital-induced nuclear translocation of CAR are impaired, leading to elevated serum bilirubin levels (68). Although CAR activity itself has not been investigated in fatty liver disease, type 2 diabetes or cholestasis, it has been proposed as a potential therapeutic target in these conditions (69–71). In this study, we found evidence that *Nr1i3* mRNA expression is downregulated and that CAR transcriptional activity is impaired in the liver during sepsis in mice, pigs and human hepatocytes from humanized-liver mice. Our results align with previous reports showing that numerous drug-metabolizing enzymes and transporters are downregulated in septic mice and pigs (38). Moreover, alterations in drug metabolism, as well as increased serum bile acid and bilirubin levels, have been observed in sepsis patients in the clinic (39, 72, 73). Nevertheless, we are currently conducting two clinical studies to collect liver biopsies from dogs with spontaneous septic peritonitis and humans with peritoneal sepsis. RNA-Seq will be performed on these samples to further validate the translatability of our findings.

Reduced HNF4α binding to the *Nr1i3* promoter and diminished *Ppara* expression has been observed in the liver during sepsis, which both contributed to the downregulation of *Nr1i3* mRNA, as found in this study. HNF4α and PPARα are known regulators of *Nr1i3* transcription, but their link with CAR in sepsis has not been described (14, 55, 56). Its reduced expression was associated with decreased levels of the histone marks H3K4me3 and H3k27ac at the *Nr1i3* promoter, both indicators of active transcription, consistent with the known role of HNF4α in recruiting histone acetyl transferases to promote a transcriptional permissive chromatin environment (74). Despite the rapid downregulation of *Nr1i3* mRNA, CAR protein levels decreased only after 24h post-CLP, indicating relative protein stability. While the half-life of *Nr1i3* mRNA is approximately 6.6h, the half-life of CAR protein remains unknown and could be investigated in primary hepatocytes in future experiments (75). Furthermore, it should be noted that CAR antibodies are often nonspecific, and some only recognize endogenous CAR when it is overexpressed, which poses a limitation for studying CAR (76). Therefore, we primarily restricted our analyses to commercial ELISA and MS.

We propose HNF4α to maintain an open chromatin state at CAR DNA binding sites and suggest that the loss of HNF4α binding during CLP promotes chromatin closure at these sites, preventing CAR from binding. To confirm this, CAR ChIP-Seq should be performed in CLP liver samples and integrated with our existing HNF4α ChIP-Seq and ATAC-Seq data from livers 8h after CLP. Nevertheless, these results indicate that both reduced *Nr1i3* mRNA expression and impaired CAR DNA binding contribute to the diminished CAR transcriptional activity observed during sepsis, and that both mechanisms are affected by HNF4α. The *Ppara* motif was also enriched at CAR DNA binding sites, suggesting that PPARα may contribute to the impaired CAR DNA binding in sepsis. However, this remains a hypothesis, as no physical interaction between CAR and PPARα has been reported, nor has a role for PPARα in enhancing CAR DNA binding been established. Nonetheless, it has been reported that the PPARα agonist fenofibrate, when combined with the CAR agonist phenobarbital, further upregulates *Cyp2b10* expression (77). Furthermore, since the DNA probe used in our *in vitro* DNA binding assay remained unchanged, other mechanisms are likely involved. Potential mechanisms include post-translational modifications of CAR or a reduced interaction with its heterodimerization partner RXR or the coactivator PGC1α. CAR has multiple phosphorylation sites in its ligand-binding and DNA-binding domains, with Thr38, a site phosphorylated by PKC, mainly implicated in regulating its nuclear translocation, while Ser202 also plays a role (78). Although little has been published about other post-translational modifications, similar to PXR, CAR activity could also be regulated by acetylation and sumoylation (79). Furthermore, fasting increases *Nr1i3* mRNA expression and activates CAR through its interaction with PGC1α (14). Other important coactivators of CAR include SRC-1 and GRIP1. Additionally, CAR can regulate transcription either as a monomer or as a heterodimer with RXR, making both PGC1α and RXR interesting targets for investigation in sepsis in the context of CAR (80).

We further showed here that CAR loss-of-function during sepsis leads to the downregulation of various hepatic metabolic genes, including those involved in monocarboxylic acid metabolism, which were further downregulated by CINPA1 treatment and correlated with lethality. This was associated with the induction of the hepatic APR. Since many APPs have anti-inflammatory and tissue-repairing functions essential for hepatic regeneration, the induction of this APR is to be considered beneficial. However, proliferation markers *Mki67* and *Pcna* remained downregulated, and ALT, a biomarker for liver failure and hepatocellular injury (64), remained elevated in CLP. This suggests that, although APPs were upregulated, there was no additional evidence of enhanced liver regeneration following CAR inhibition. The role of CAR in promoting hepatocyte proliferation has been previously described in the literature (18–20). Furthermore, we previously published that HNF4α is essential for a proper APR, as IL6-mediated induction of the APR was reduced in HNF4α KO mice, and APPs such as SAA and SAP were downregulated in the plasma of these KO mice, where hepatic CAR protein is also absent, as observed in this study (9). In that paper, we also showed that APP levels were higher in a sublethal CLP compared to a lethal CLP, suggesting a correlation between APP levels and lethality in sepsis. Since HNF4α promotes *Nr1i3* transcription and CAR activation downregulates the expression of APR genes in the liver, this finding appears somewhat contradictory. However, other studies have found contradictory results, as despite HNF4α regulates *Nr1i3* mRNA expression, HNF4α and CAR often have opposite effects on gene transcription. For example, CAR inhibits gluconeogenesis, while HNF4α stimulates it (81). Additionally, CAR suppresses *Ppara* expression, whereas HNF4α promotes it (12, 82). This is because CAR and HNF4α frequently bind in close proximity to one another, as shown by public ChIP-Seq data and observed in this study, where the *Hnf4a* motif was enriched at CAR DNA binding sites. They do not prevent each other’s binding to DNA. They even enhance each other’s binding, likely through chromatin remodeling, a process known as assisted loading (83). This is further supported by our observation that CAR DNA binding sites were less accessible in the absence of hepatic HNF4α. In fact, they compete for the same coregulators, such as PGC1α and GRIP-1, and genes also compete for shared local enhancers, so that the activation of one gene may repress another that relies on the same enhancers (83). This type of gene regulation likely occurs to enable a controlled response to environmental changes and prevent the overactivation of any single pathway.

CAR may also indirectly suppress the APR by inhibiting IL6 production. As noted earlier, IL6 is a proinflammatory cytokine and a marker of systemic inflammation, playing a key role in APP production and liver regeneration (61, 62). In our study, plasma IL6 and SAA levels were both elevated upon CAR inhibition with CINPA1 in CLP. Little is known about CAR as a regulator of immune cell function, except that it controls MDR1 expression in CD4^+^ effector T cells to prevent bile-acid induced toxicity and inflammation in the small intestine (84). However, many other nuclear receptors, including HNF4α, PPARα and PXR, have been reported to suppress immune activation, while their expression is downregulated by proinflammatory cytokines or LPS (85–87). Forced expression of HNF4α attenuates RelA (p65) expression and nuclear translocation, thereby impairing NFκB activation (86). PPARα inhibits inflammatory signaling mediated by NFκB, AP-1 and STATs, promotes the catabolism of inflammatory lipids and may regulate the expression of toll-like receptors (TLRs) and proteins involved in TLR signaling (85). In addition, PXR, considered functionally redundant with CAR, suppresses NFκB activation and regulates the expression of TLRs and NLRP3, a key component of the inflammasome (87). Consistent with our findings on CAR and IL6, plasma IL6 levels were elevated in Hnf4a^Liver-i-KO^ and PXR KO mice, and decreased following treatment with the PPARα agonist pemafibrate, confirming their suppressive roles in systemic inflammation (8, 9, 88). Therefore, their downregulation or functional impairment during sepsis may contribute to an uncontrolled immune response.

Despite our observation that CAR loss-of-function during sepsis leads to the downregulation of various hepatic metabolic genes, including those involved in the metabolism of bile acids and fatty acids, as well as genes from the TCA cycle, additional metabolic data, such as the measurement of bile acids and bilirubin, would further strengthen these findings. Bile acids are synthesized from cholesterol in the liver and are normally secreted into the bile, which is released into the small intestine to aid in fat absorption and digestion. However, when they accumulate in the liver and bloodstream, as in cholestasis due to impaired bile flow, they can damage cell membranes, cause liver injury and trigger hepatic inflammation, which over time can lead to fibrosis, cirrhosis, and eventually liver failure (89). Similarly, bilirubin, a waste product formed from the degradation of hemoglobin, normally excreted into the bile, can become toxic at high levels in the blood, a condition known as hyperbilirubinemia or jaundice, leading to neurotoxicity and oxidative stress (90). Both cholestasis and hyperbilirubinemia are associated with increased mortality in sepsis (72, 73). Furthermore, CAR plays a significant role in energy homeostasis, which is profoundly disrupted in sepsis, characterized by reduced gluconeogenesis, hypoglycemia, impaired β-oxidation, and hepatic steatosis (7, 8). However, CAR activation has been shown to decrease gluconeogenesis and serum glucose levels while enhancing mitochondrial metabolism and reducing lactate production in HepaRG cells (81, 91). In this regard, sepsis-induced downregulation of CAR may serve as a mechanism to prevent further inhibition of gluconeogenesis and the subsequent decline in serum glucose levels. The role of CAR in lipid metabolism remains controversial and may depend on the specific model and CAR agonist used, making it difficult to speculate on how CAR activation might affect lipid metabolism in sepsis (69, 82, 92, 93).

While CINPA1-mediated CAR inhibition increased sepsis lethality, highlighting the importance of physiological CAR in this condition, TCPOBOP treatment failed to provide protection. Studies have reported that in CD-1 mice, 1 day after a single dose of TCPOBOP, genes involved in lipid metabolism, xenobiotic processing, and oxidative pathways are upregulated. In contrast, after 2 weeks, secondary and indirect CAR target genes, which are involved in immune response, macrophage activation, and the production of cytokines and reactive oxygen species, are upregulated. Upstream regulators associated with pro-inflammatory responses and hepatocellular carcinoma (HCC) progression are also activated, accompanied by hepatic cholesterol and lipid accumulation, as well as an increase in ALT levels in the blood, indicating hepatocyte damage. These findings suggest that prolonged TCPOBOP exposure may lead to metabolic dysfunction-associated steatotic liver disease (MASLD) and, ultimately, HCC (94). Based on these findings, it could be hypothesized that TCPOBOP initially protects by promoting CAR activity but later contributes to increased lethality. However, no significant difference in lethality was observed with TCPOBOP treatment throughout the entire survival course. Future experiments could explore the use of other CAR agonists, or to use TCPOBOP in extremely fast or slow sepsis models to study this hypothesis. In terms of other CAR ligands, most have been reported to be carcinogenic in mice, not in humans. In human liver cancer, CAR is even considered a tumor-suppressor (95). Furthermore, since *Nr1i3* mRNA expression levels are reduced in sepsis, compromising responsiveness to TCPOBOP, targeting upstream of CAR, as demonstrated with NCT, seems more rational. With NCT, we were able to prevent reduced HNF4α binding to the *Nr1i3* promoter, thereby increasing *Nr1i3* mRNA expression, enhancing CAR transcriptional activity, and contributing to its protective effects during sepsis.

We conclude that *Nr1i3* mRNA expression is decreased and CAR DNA binding is impaired, thereby reducing its transcriptional activity in the liver during sepsis. Both HNF4α and PPARα contribute to its transcriptional downregulation, while HNF4α also regulates the DNA binding capacity of CAR by modulating chromatin accessibility at its binding sites. However, other mechanisms, such as post-translational modifications of CAR or reduced interactions with RXR or PGC1α, are also likely to contribute to its impaired DNA binding. Although endogenous CAR activity is essential during sepsis, TCPOBOP-induced activation fails to improve survival, likely due to sepsis-associated TCPOBOP resistance. In that regard, activating HNF4α to enhance *Nr1i3* transcription and CAR activity may be a more beneficial therapeutic approach in sepsis. Nevertheless, it might still be possible that TCPOBOP protects in mice where endogenous CAR activity is nearly completely lost. Furthermore, CAR loss-of-function in sepsis impairs hepatic metabolism, particularly monocarboxylic acid, bile acid, bilirubin and lipid metabolism, while inducing the hepatic APR. However, additional metabolic data, such as CYP enzyme activity and plasma bile acid, bilirubin, glucose, and FFA levels, along with the generation of CAR KO mice, could provide further insights into the function of CAR during sepsis and help us understand the mechanisms by which CAR inhibition with CINPA1 increases lethality. Although no signs of improved liver regeneration were observed upon CAR inhibition in sepsis, this could be further investigated through time-course studies or staining for proliferation markers. Furthermore, since several CAR target genes, including *Cyp2b10*, correlated with lethality, hepatic CAR activity, or hepatic *Nr1i3* or *Cyp2b10* mRNA expression, could be explored as biomarkers in sepsis in the future, with plasma bilirubin and bile acid levels as potential surrogates.

## Data availability statement

RNA-Seq data have been deposited at the National Center for Biotechnology Information Gene Expression Omnibus (GEO) public database (http://www.ncbi.nlm.nih.gov/geo/) and are publicly available as of the date of publication under accession number GSE294204. MS proteomics data have been deposited to the ProteomeXchange Consortium via the PRIDE partner repository (96, 97) with the dataset identifier PXD062961. Data are publicly available as of the date of publication. This paper also makes use of publicly available datasets, including: RNA-Seq data from liver 8h and 24h after CLP (GEO: GSE160795 & GSE160830); RNA-Seq data from liver 10h after CLP (GEO: GSE139484); CAR ChIP-Seq data from liver 2h after the final TCPOBOP injection (GEO: GE112199); HNF4α ChIP-Seq data from liver 8h after CLP (GEO: GSE245682); ATAC-Seq data from liver 8h after CLP (GEO: GSE244821); H3K4me3 & H3K27ac ChIP-Seq data from liver 8h after CLP (GEO: GSE260577); HNF4α KO ATAC-Seq data from liver (ArrayExpress: E-MTAB-10266); RNA-Seq data from liver after TCPOBOP injection in CAR KO mice (GEO: GSE40120), and RNA-Seq data from septic pigs (GEO: GSE218636).
