# Supplementary material for: HNF4α contributes to hepatic CAR dysfunction in polymicrobial sepsis
Source: Front Immunol. 2025 Aug 19;16:1625104. doi: 10.3389/fimmu.2025.1625104 (PMC12401704; doi:10.3389/fimmu.2025.1625104)
Supplement: Supplementary file 1 [file Presentation1.pptx]

## Slide 1
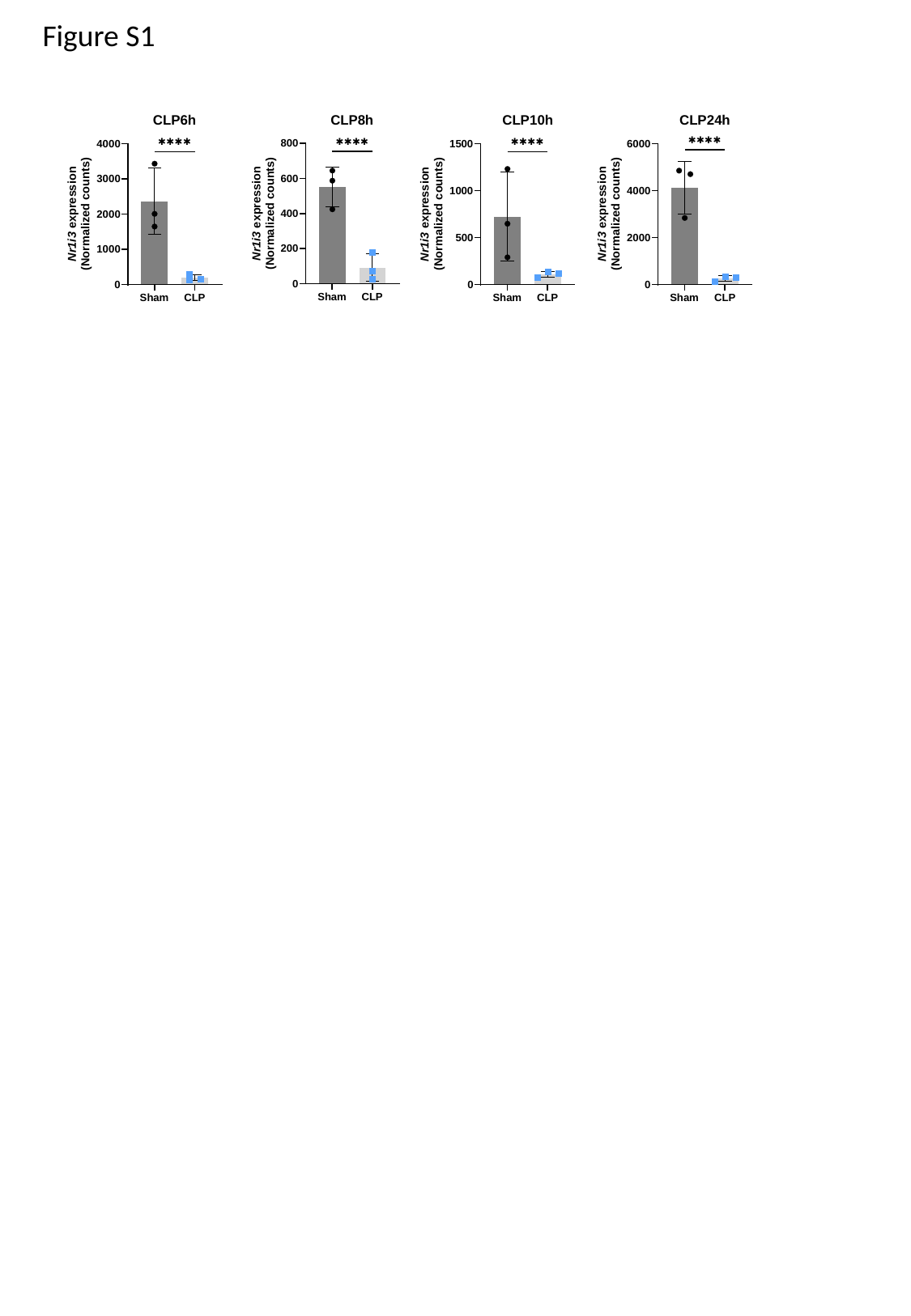

Figure S1

## Slide 2
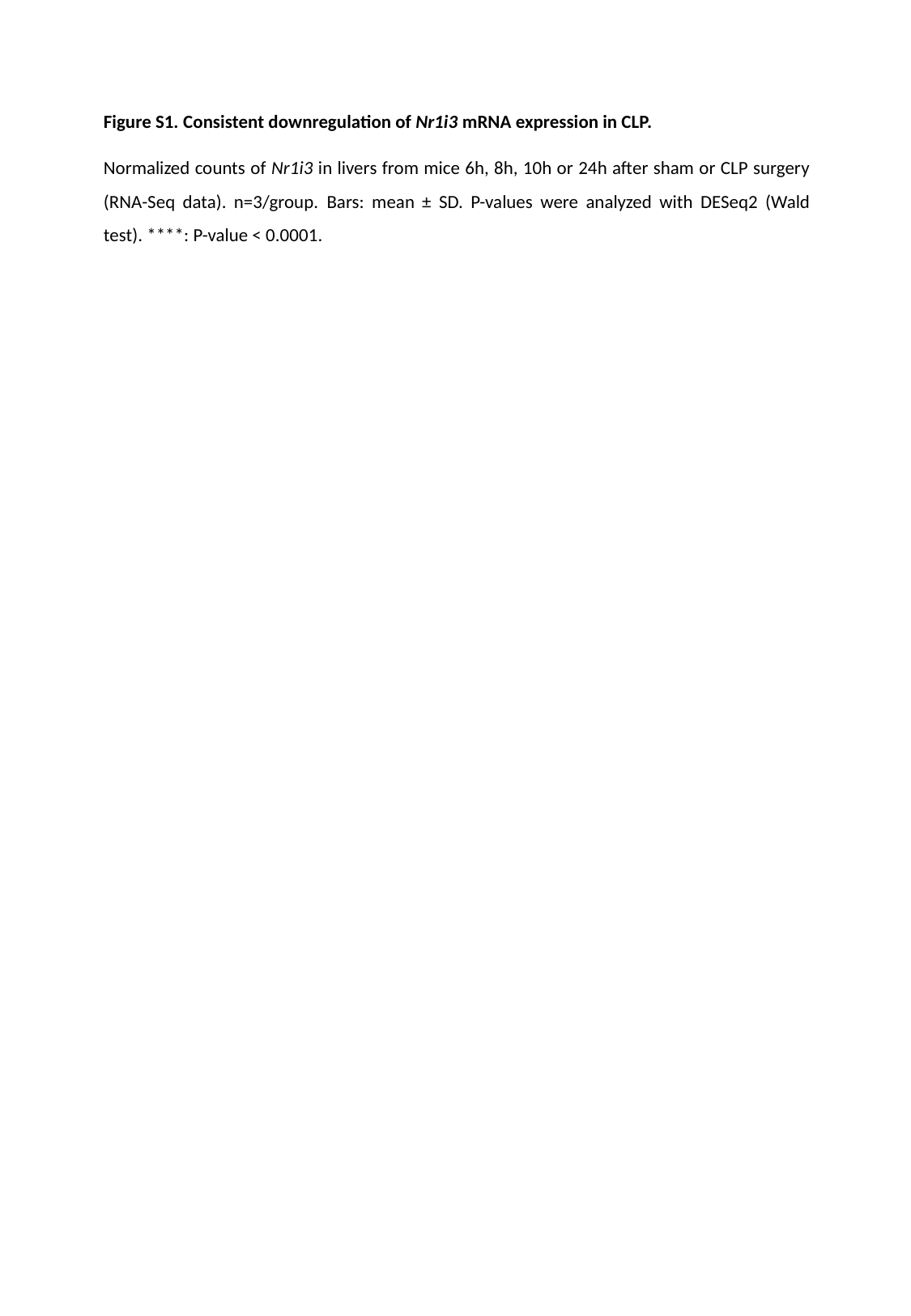

Figure S1. Consistent downregulation of Nr1i3 mRNA expression in CLP.
Normalized counts of Nr1i3 in livers from mice 6h, 8h, 10h or 24h after sham or CLP surgery (RNA-Seq data). n=3/group. Bars: mean ± SD. P-values were analyzed with DESeq2 (Wald test). ****: P-value < 0.0001.

## Slide 3
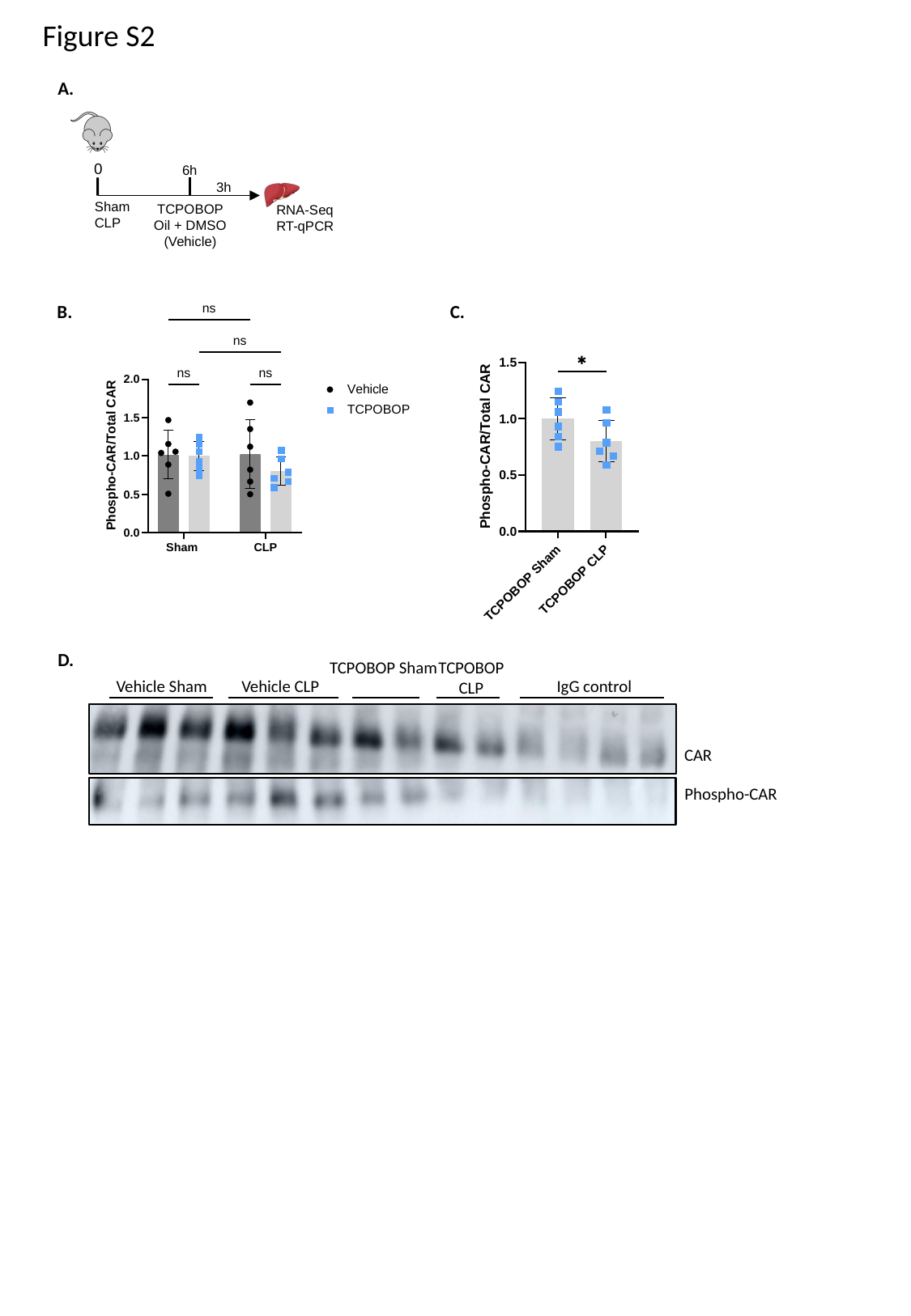

Figure S2
A.
0
6h
3h
Sham
CLP
TCPOBOP Oil + DMSO (Vehicle)
RNA-Seq
RT-qPCR
C.
B.
D.
TCPOBOP Sham
TCPOBOP CLP
Vehicle Sham
Vehicle CLP
IgG control
CAR
Phospho-CAR

## Slide 4
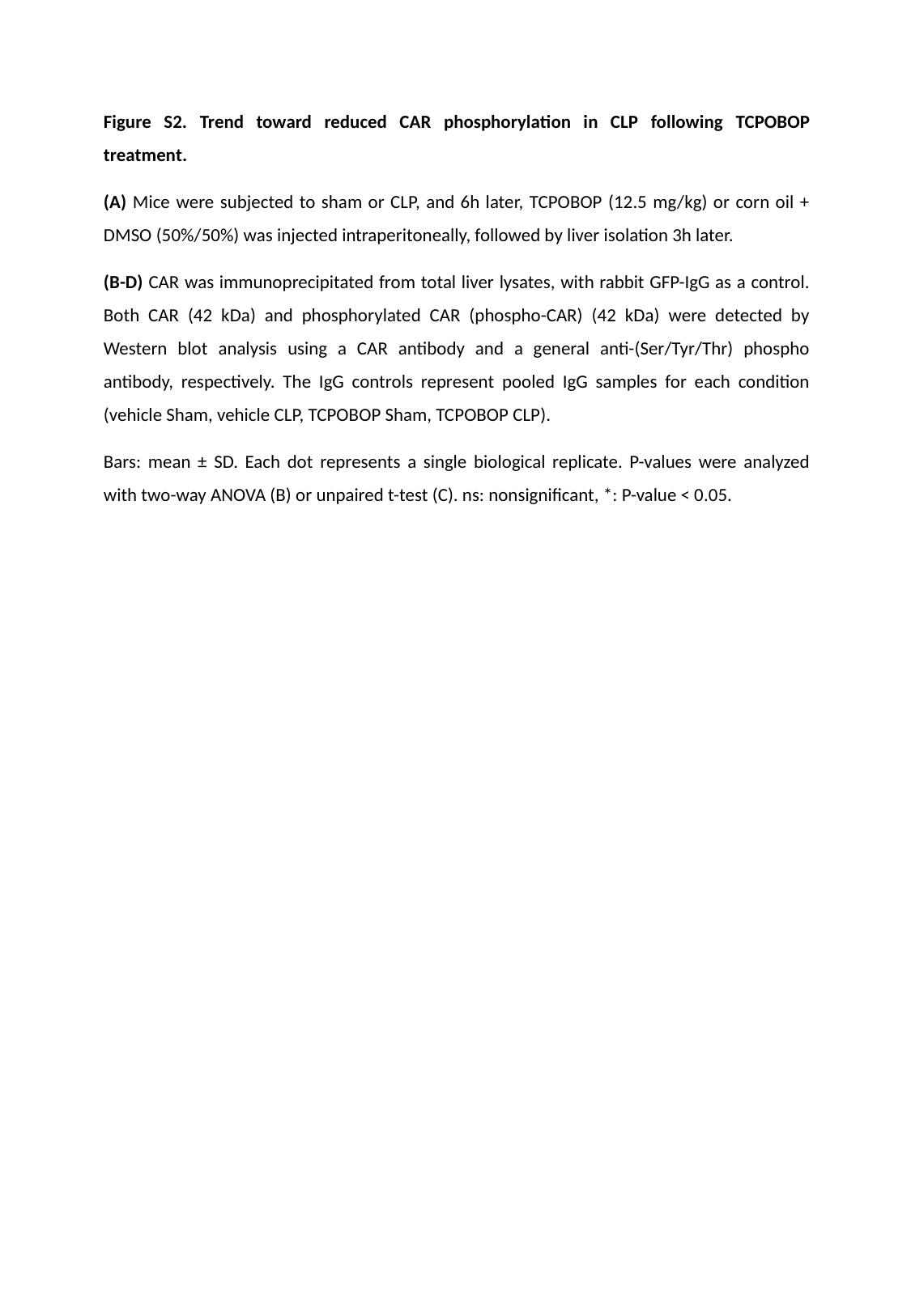

Figure S2. Trend toward reduced CAR phosphorylation in CLP following TCPOBOP treatment.
(A) Mice were subjected to sham or CLP, and 6h later, TCPOBOP (12.5 mg/kg) or corn oil + DMSO (50%/50%) was injected intraperitoneally, followed by liver isolation 3h later.
(B-D) CAR was immunoprecipitated from total liver lysates, with rabbit GFP-IgG as a control. Both CAR (42 kDa) and phosphorylated CAR (phospho-CAR) (42 kDa) were detected by Western blot analysis using a CAR antibody and a general anti-(Ser/Tyr/Thr) phospho antibody, respectively. The IgG controls represent pooled IgG samples for each condition (vehicle Sham, vehicle CLP, TCPOBOP Sham, TCPOBOP CLP).
Bars: mean ± SD. Each dot represents a single biological replicate. P-values were analyzed with two-way ANOVA (B) or unpaired t-test (C). ns: nonsignificant, *: P-value < 0.05.

## Slide 5
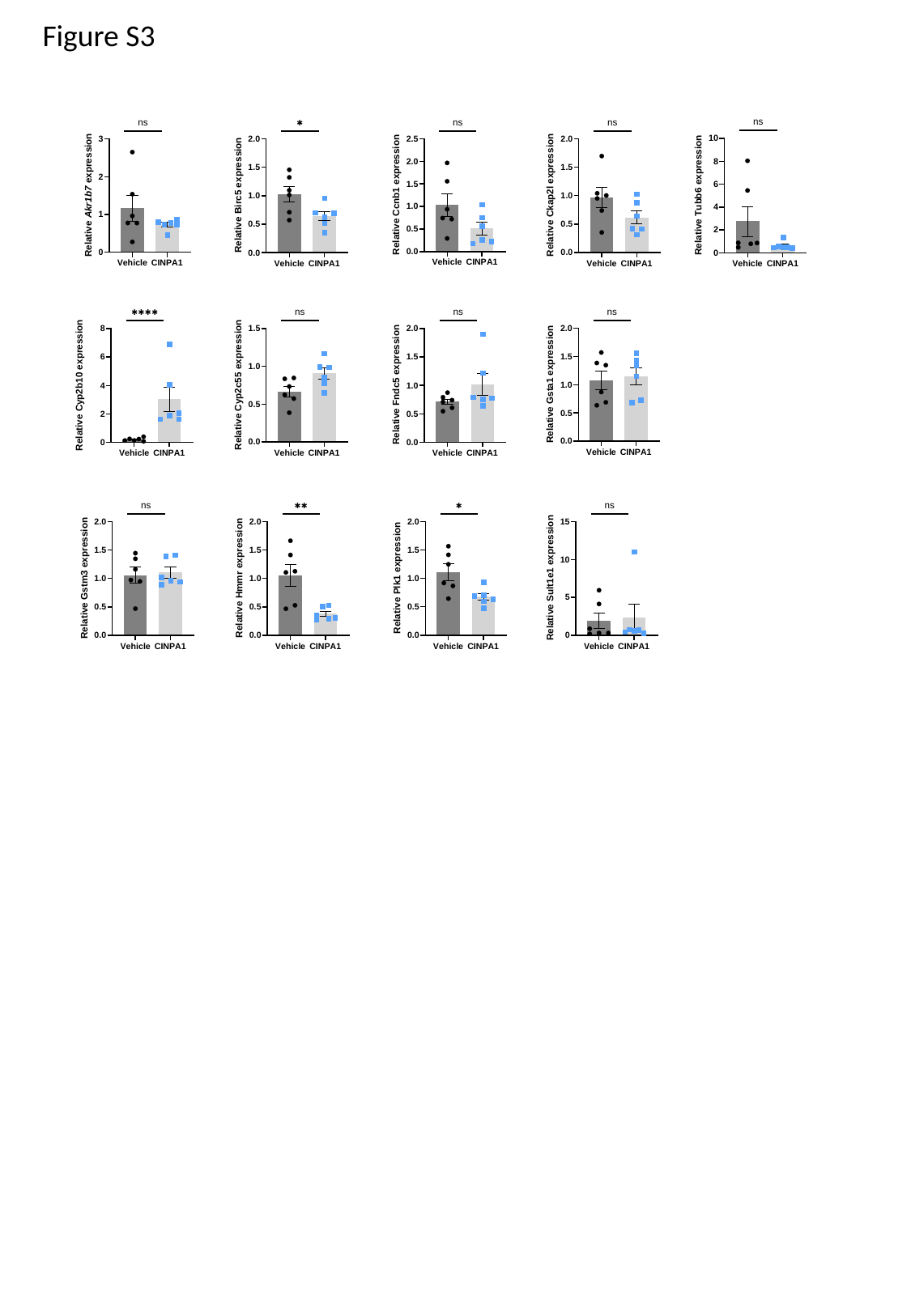

Figure S3

## Slide 6
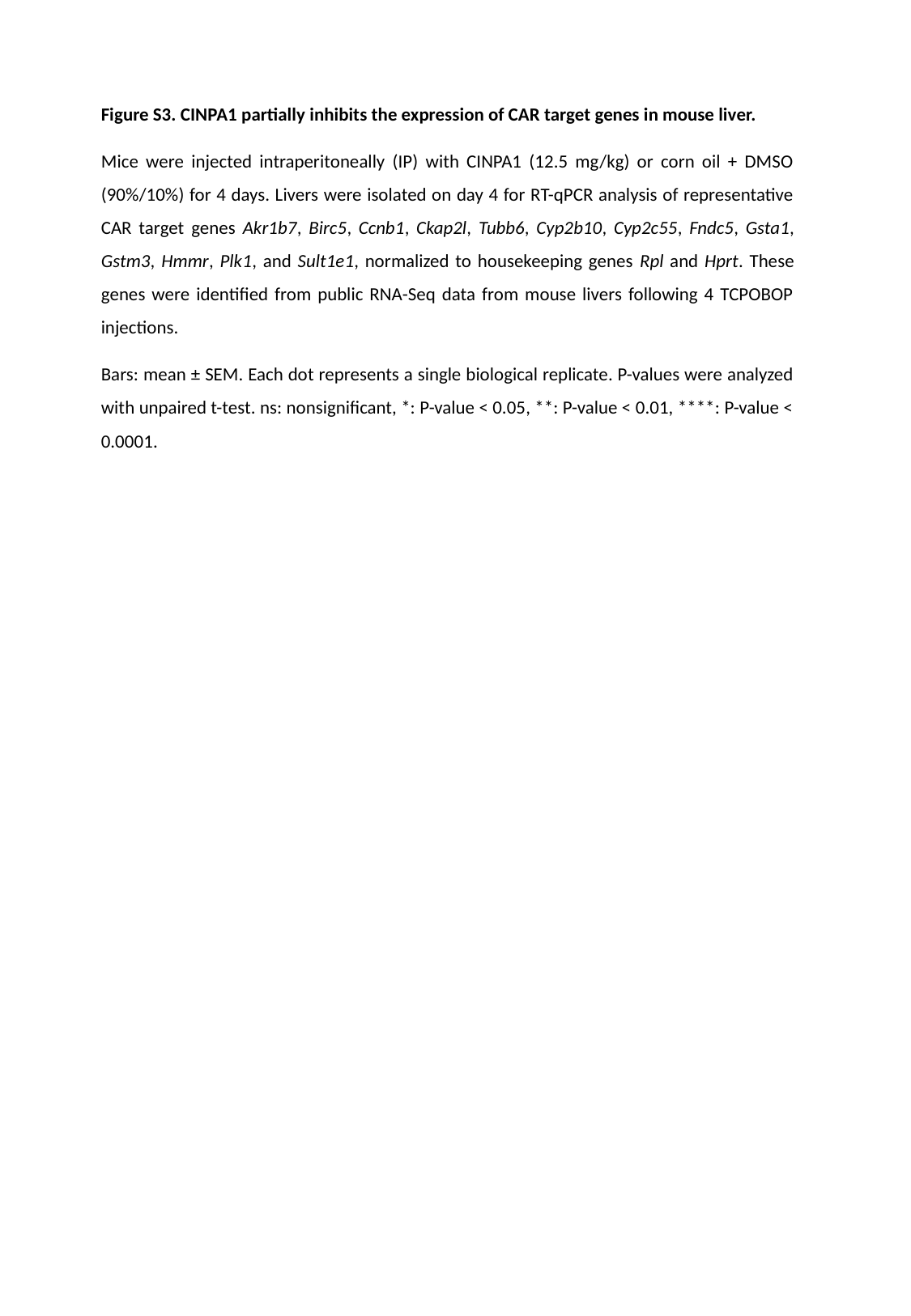

Figure S3. CINPA1 partially inhibits the expression of CAR target genes in mouse liver.
Mice were injected intraperitoneally (IP) with CINPA1 (12.5 mg/kg) or corn oil + DMSO (90%/10%) for 4 days. Livers were isolated on day 4 for RT-qPCR analysis of representative CAR target genes Akr1b7, Birc5, Ccnb1, Ckap2l, Tubb6, Cyp2b10, Cyp2c55, Fndc5, Gsta1, Gstm3, Hmmr, Plk1, and Sult1e1, normalized to housekeeping genes Rpl and Hprt. These genes were identified from public RNA-Seq data from mouse livers following 4 TCPOBOP injections.
Bars: mean ± SEM. Each dot represents a single biological replicate. P-values were analyzed with unpaired t-test. ns: nonsignificant, *: P-value < 0.05, **: P-value < 0.01, ****: P-value < 0.0001.

## Slide 7
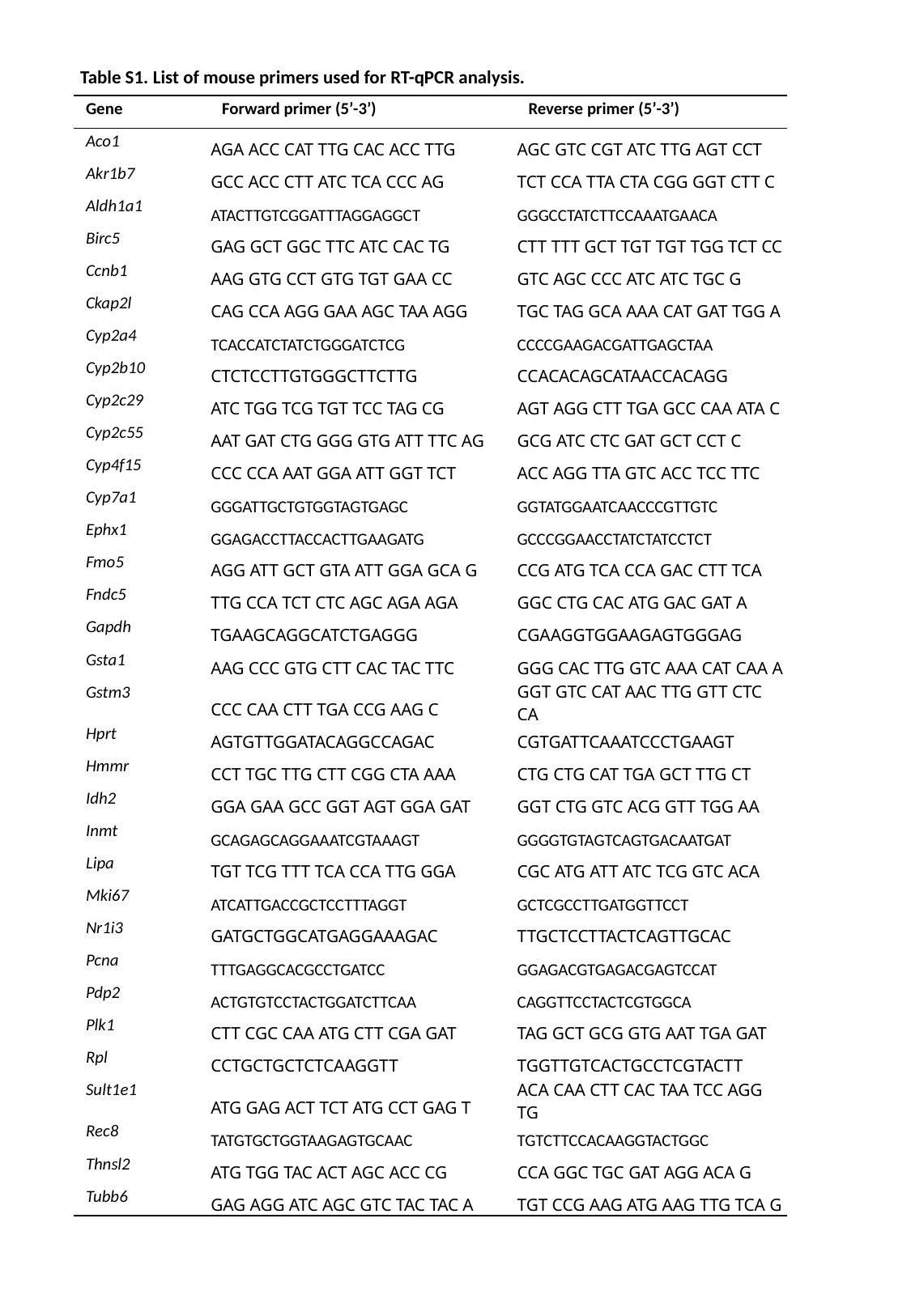

Table S1. List of mouse primers used for RT-qPCR analysis.
| Gene | Forward primer (5’-3’) | Reverse primer (5’-3’) |
| --- | --- | --- |
| Aco1 | AGA ACC CAT TTG CAC ACC TTG | AGC GTC CGT ATC TTG AGT CCT |
| Akr1b7 | GCC ACC CTT ATC TCA CCC AG | TCT CCA TTA CTA CGG GGT CTT C |
| Aldh1a1 | ATACTTGTCGGATTTAGGAGGCT | GGGCCTATCTTCCAAATGAACA |
| Birc5 | GAG GCT GGC TTC ATC CAC TG | CTT TTT GCT TGT TGT TGG TCT CC |
| Ccnb1 | AAG GTG CCT GTG TGT GAA CC | GTC AGC CCC ATC ATC TGC G |
| Ckap2l | CAG CCA AGG GAA AGC TAA AGG | TGC TAG GCA AAA CAT GAT TGG A |
| Cyp2a4 | TCACCATCTATCTGGGATCTCG | CCCCGAAGACGATTGAGCTAA |
| Cyp2b10 | CTCTCCTTGTGGGCTTCTTG | CCACACAGCATAACCACAGG |
| Cyp2c29 | ATC TGG TCG TGT TCC TAG CG | AGT AGG CTT TGA GCC CAA ATA C |
| Cyp2c55 | AAT GAT CTG GGG GTG ATT TTC AG | GCG ATC CTC GAT GCT CCT C |
| Cyp4f15 | CCC CCA AAT GGA ATT GGT TCT | ACC AGG TTA GTC ACC TCC TTC |
| Cyp7a1 | GGGATTGCTGTGGTAGTGAGC | GGTATGGAATCAACCCGTTGTC |
| Ephx1 | GGAGACCTTACCACTTGAAGATG | GCCCGGAACCTATCTATCCTCT |
| Fmo5 | AGG ATT GCT GTA ATT GGA GCA G | CCG ATG TCA CCA GAC CTT TCA |
| Fndc5 | TTG CCA TCT CTC AGC AGA AGA | GGC CTG CAC ATG GAC GAT A |
| Gapdh | TGAAGCAGGCATCTGAGGG | CGAAGGTGGAAGAGTGGGAG |
| Gsta1 | AAG CCC GTG CTT CAC TAC TTC | GGG CAC TTG GTC AAA CAT CAA A |
| Gstm3 | CCC CAA CTT TGA CCG AAG C | GGT GTC CAT AAC TTG GTT CTC CA |
| Hprt | AGTGTTGGATACAGGCCAGAC | CGTGATTCAAATCCCTGAAGT |
| Hmmr | CCT TGC TTG CTT CGG CTA AAA | CTG CTG CAT TGA GCT TTG CT |
| Idh2 | GGA GAA GCC GGT AGT GGA GAT | GGT CTG GTC ACG GTT TGG AA |
| Inmt | GCAGAGCAGGAAATCGTAAAGT | GGGGTGTAGTCAGTGACAATGAT |
| Lipa | TGT TCG TTT TCA CCA TTG GGA | CGC ATG ATT ATC TCG GTC ACA |
| Mki67 | ATCATTGACCGCTCCTTTAGGT | GCTCGCCTTGATGGTTCCT |
| Nr1i3 | GATGCTGGCATGAGGAAAGAC | TTGCTCCTTACTCAGTTGCAC |
| Pcna | TTTGAGGCACGCCTGATCC | GGAGACGTGAGACGAGTCCAT |
| Pdp2 | ACTGTGTCCTACTGGATCTTCAA | CAGGTTCCTACTCGTGGCA |
| Plk1 | CTT CGC CAA ATG CTT CGA GAT | TAG GCT GCG GTG AAT TGA GAT |
| Rpl | CCTGCTGCTCTCAAGGTT | TGGTTGTCACTGCCTCGTACTT |
| Sult1e1 | ATG GAG ACT TCT ATG CCT GAG T | ACA CAA CTT CAC TAA TCC AGG TG |
| Rec8 | TATGTGCTGGTAAGAGTGCAAC | TGTCTTCCACAAGGTACTGGC |
| Thnsl2 | ATG TGG TAC ACT AGC ACC CG | CCA GGC TGC GAT AGG ACA G |
| Tubb6 | GAG AGG ATC AGC GTC TAC TAC A | TGT CCG AAG ATG AAG TTG TCA G |

## Slide 8
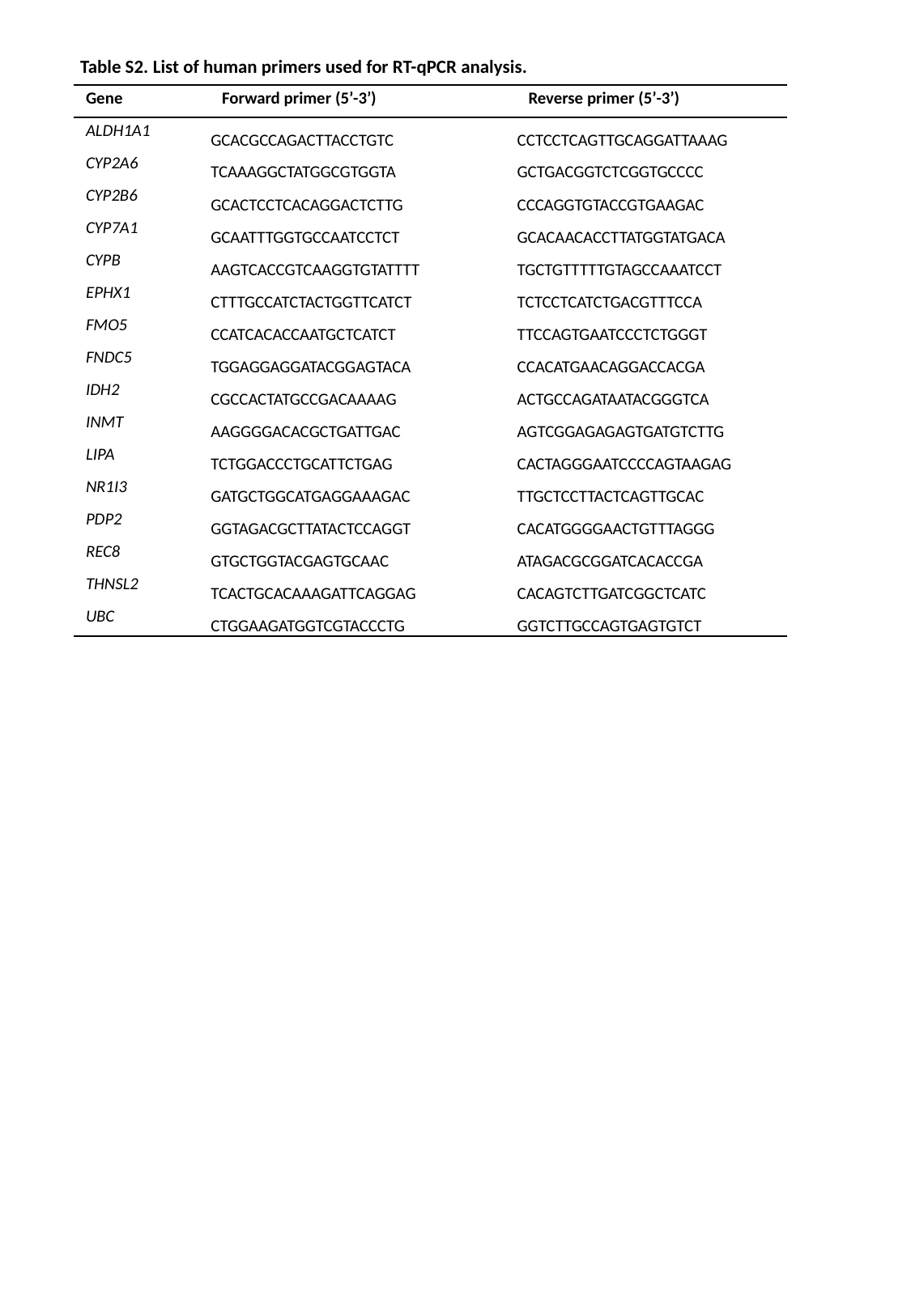

Table S2. List of human primers used for RT-qPCR analysis.
| Gene | Forward primer (5’-3’) | Reverse primer (5’-3’) |
| --- | --- | --- |
| ALDH1A1 | GCACGCCAGACTTACCTGTC | CCTCCTCAGTTGCAGGATTAAAG |
| CYP2A6 | TCAAAGGCTATGGCGTGGTA | GCTGACGGTCTCGGTGCCCC |
| CYP2B6 | GCACTCCTCACAGGACTCTTG | CCCAGGTGTACCGTGAAGAC |
| CYP7A1 | GCAATTTGGTGCCAATCCTCT | GCACAACACCTTATGGTATGACA |
| CYPB | AAGTCACCGTCAAGGTGTATTTT | TGCTGTTTTTGTAGCCAAATCCT |
| EPHX1 | CTTTGCCATCTACTGGTTCATCT | TCTCCTCATCTGACGTTTCCA |
| FMO5 | CCATCACACCAATGCTCATCT | TTCCAGTGAATCCCTCTGGGT |
| FNDC5 | TGGAGGAGGATACGGAGTACA | CCACATGAACAGGACCACGA |
| IDH2 | CGCCACTATGCCGACAAAAG | ACTGCCAGATAATACGGGTCA |
| INMT | AAGGGGACACGCTGATTGAC | AGTCGGAGAGAGTGATGTCTTG |
| LIPA | TCTGGACCCTGCATTCTGAG | CACTAGGGAATCCCCAGTAAGAG |
| NR1I3 | GATGCTGGCATGAGGAAAGAC | TTGCTCCTTACTCAGTTGCAC |
| PDP2 | GGTAGACGCTTATACTCCAGGT | CACATGGGGAACTGTTTAGGG |
| REC8 | GTGCTGGTACGAGTGCAAC | ATAGACGCGGATCACACCGA |
| THNSL2 | TCACTGCACAAAGATTCAGGAG | CACAGTCTTGATCGGCTCATC |
| UBC | CTGGAAGATGGTCGTACCCTG | GGTCTTGCCAGTGAGTGTCT |
